# A nuclear CobW/WW-domain factor represses the CO_2_-concentrating mechanism in the green alga *Chlamydomonas reinhardtii*

**DOI:** 10.1073/pnas.2518136123

**Published:** 2026-02-04

**Authors:** Daisuke Shimamura, Junko Yasuda, Yosuke Yamahara, Hirobumi Nakano, Shin-Ichiro Ozawa, Ryutaro Tokutsu, Ayumi Yamagami, Tomonao Matsushita, Yuichiro Takahashi, Takeshi Nakano, Hideya Fukuzawa, Takashi Yamano

**Affiliations:** ^a^Graduate School of Biostudies, Division of Integrated Life Science, Kyoto University, Kyoto 606-8502, Japan; ^b^RIKEN Center for Sustainable Resource Science, Yokohama 230-0045, Japan; ^c^Institute of Plant Science and Resources, Okayama University, Kurashiki 710-0046, Japan; ^d^Research Institute for Interdisciplinary Science, Okayama University, Okayama 700-8530, Japan; ^e^Graduate School of Science, Division of Biological Sciences, Kyoto University, Kyoto 606-8502, Japan; ^f^School of Veterinary Medicine, Kitasato University, Sagamihara 252-0329, Kanagawa, Japan; ^g^Center for Higher Educational Development, Kyoto Women’s University, Kyoto 605-8501, Japan; ^h^Center for Living Systems Information Science, Kyoto University, Kyoto 606-8501, Japan

**Keywords:** carbonic anhydrase, *Chlamydomonas reinhardtii*, CO_2_-concentrating mechanism, photosynthesis, pyrenoid

## Abstract

Microalgae induce a CO_2_-concentrating mechanism (CCM) to maintain photosynthesis when CO_2_ is limited. Because this system consumes a substantial portion of photosynthetically generated ATP, its suppression when CO_2_ levels rise is critical for energy balance, yet the underlying mechanism remains unclear. Here, we identify a nuclear repressor of the CCM in the green alga *Chlamydomonas reinhardtii*. A pull-down screen for interacting partners of the master activator CCM1/CIA5 revealed an uncharacterized protein that tightly associates with CCM1. This protein, CCM1-binding protein 1 (CBP1), combines a CobW/CobW_C GTP-binding metallochaperone module with a WW-domain characteristic of protein–protein interactions. CBP1 colocalizes and interacts with CCM1 in the nucleus regardless of CO_2_ conditions. Disruption of CBP1 does not affect growth or CCM induction under CO_2_ limitation but derepresses 27 of 41 CCM1-dependent low-CO_2_ inducible genes under high-CO_2_ conditions. These include the periplasmic and intracellular carbonic anhydrases (CAH1 and LCIB) and inorganic carbon transporters/channels (LCIA, LCI1, BST1, and BST3). Consistently, *cbp1* mutants accumulate CAH1 and LCIB proteins and exhibit 40% higher inorganic carbon affinity under high-CO_2_ conditions; this phenotype is rescued by CBP1 complementation or by acetazolamide treatment. Crucially, *cbp1* mutants exhibit significant growth delays under high-CO_2_ conditions, especially when light is limiting, providing direct evidence that CBP1-mediated repression is essential for energy conservation. Thus, CBP1 prevents unnecessary CCM activity when CO_2_ is abundant, acting upstream of both transporter/channel and carbonic anhydrase modules. Our findings suggest a regulatory mechanism potentially linking zinc-dependent protein chemistry to CCM gene repression, providing insights into energy-efficient CO_2_ sensing in aquatic photosynthetic organisms.

## Significance

Algae flourish by activating a CO_2_-concentrating mechanism (CCM) when dissolved CO_2_ is limited but must deactivate it when CO_2_ levels increase to conserve energy. We have identified the nuclear protein that represses CCM gene expression under high CO_2_ conditions in the model green alga. Deletion of this regulator leads to the derepression of CCM transporters and enzymes, maintaining CCM activity even under high-CO_2_ conditions and imposing an unnecessary energetic burden. This finding illuminates how algae balance energy consumption with carbon capture and offers a target for engineering strains that fix CO_2_ more efficiently for biofuel production or climate-mitigation technologies.

Efficient CO_2_ sensing enables photosynthetic organisms to maximize carbon acquisition while minimizing resource expenditure. In terrestrial plants, stomata regulate their aperture in response to environmental CO_2_, balancing photosynthesis with transpiration (1). A recently identified guard-cell complex comprising the Raf-like kinase HT1 and MAP kinases MPK12/MPK4 now provides a molecular model for CO_2_/HCO_3_^–^ sensing (2). These discoveries highlight the evolutionary diversity of CO_2_-responsive pathways.

Aquatic photoautotrophs encounter chronic inorganic carbon (Ci) limitation because dissolved CO_2_ rapidly converts to HCO_3_^–^. Many microalgae, including marine diatoms and freshwater algae, therefore employ a CO_2_-concentrating mechanism (CCM) that utilizes Ci transporters and carbonic anhydrases (CAs) to accumulate CO_2_ around the photosynthetic CO_2_-fixing enzyme, Ribulose-1,5-bisphosphate carboxylase/oxygenase (Rubisco), enhancing CO_2_ fixation (3, 4). Despite being the primary carbon fixation enzyme, Rubisco has relatively low affinity and specificity for CO_2_, making CCM essential under CO_2_-limiting conditions (5). The green alga *Chlamydomonas reinhardtii* (hereafter *Chlamydomonas*) strongly induces Ci transporters, CAs, and regulatory proteins such as low-CO_2_ response regulator 1 (LCR1) when CO_2_ becomes limiting (6, 7).

Central to CCM regulation in *Chlamydomonas* is CCM1/CIA5 (hereafter CCM1), a zinc-binding protein identified independently by two research groups (8, 9). Loss-of-function mutants of CCM1 fail to induce essential CCM genes under CO_2_-limiting conditions (6), resulting in severe growth defects. Genome-wide analyses further reveal that many CCM1-dependent genes respond to additional factors beyond CO_2_ concentration, such as light intensity, photoperiod, and nitrogen availability (10–12). Although CCM1 contains two Zn^2+^-binding sites (13, 14), direct DNA binding has not been demonstrated, suggesting that CCM1 operates in conjunction with other nuclear factors.

In contrast, mechanisms that deactivate the CCM when CO_2_ again becomes abundant remain poorly characterized. In cyanobacteria, the LysR regulators NdhR/CcmR and CmpR repress Ci-transporter operons under high CO_2_ (15, 16). A CREB/ATF-type bZIP factor performs a similar function in the diatom *Phaeodactylum tricornutum* (17). In green algae, several mutants with impaired CCM induction have been described, yet no dedicated nuclear repressor has been confirmed. This gap is significant because CCM operation can consume a substantial portion of photosynthetically generated ATP, as demonstrated by direct energy-budget measurements (18) and detailed cost evaluations (19, 20).

Metal-dependent transcriptional switches represent a plausible mechanism for such control. Members of the COG0523 GTPase family, including plant ZNG1/2, deliver Zn^2+^ to target proteins and modulate gene expression according to zinc status (21–23). Similar Zn-based sensors regulate diverse stress pathways in higher plants—for example, the bZIP19/23 system that activates ZIP4 under Zn deficiency (24). Since CCM1 itself coordinates Zn^2+^ (13), it is conceivable that changes in intracellular Zn availability could rapidly trigger a conformational change in CCM1, modulating its interaction with regulatory partners and thus providing a quick molecular switch under high CO_2_ conditions, similar to known Zn-regulated transcription factors (25).

In this study, we tested the hypothesis that a nuclear factor binds CCM1 and inhibits CCM gene expression under high CO_2_. A pull-down screen indeed identified a CobW/WW-domain protein that we name CCM1-binding protein 1 (CBP1). We demonstrate that CBP1 associates with CCM1 in vivo, localizes to the nucleus, and is specifically required to repress the low-affinity CCM program when CO_2_ is abundant. Our findings reveal a layer of CCM regulation that links zinc metabolism to energy conservation in aquatic phototrophs, addressing a regulatory aspect previously overlooked in algal CCM studies.

## Results

### Identification of the CCM1-Binding Proteins.

To identify proteins that physically interact with the master regulator CCM1, we performed a pull-down assay using *C. reinhardtii* cells expressing FLAG-tagged CCM1. Specifically, we generated a transgenic strain, C16(*ccm1*):*CCM1-FLAG* #2 (CF-2), by introducing a CCM1-FLAG construct into the *ccm1* mutant strain C16 (8) (*SI Appendix*, Fig. S1*A*). We chose heterotrophic conditions for cell growth to minimize background expression of CCM-related genes and to stabilize CCM1 expression for immunoprecipitation. We then compared immunoprecipitates obtained from the parental strain (5D) and CF-2.

**Fig. 1.**
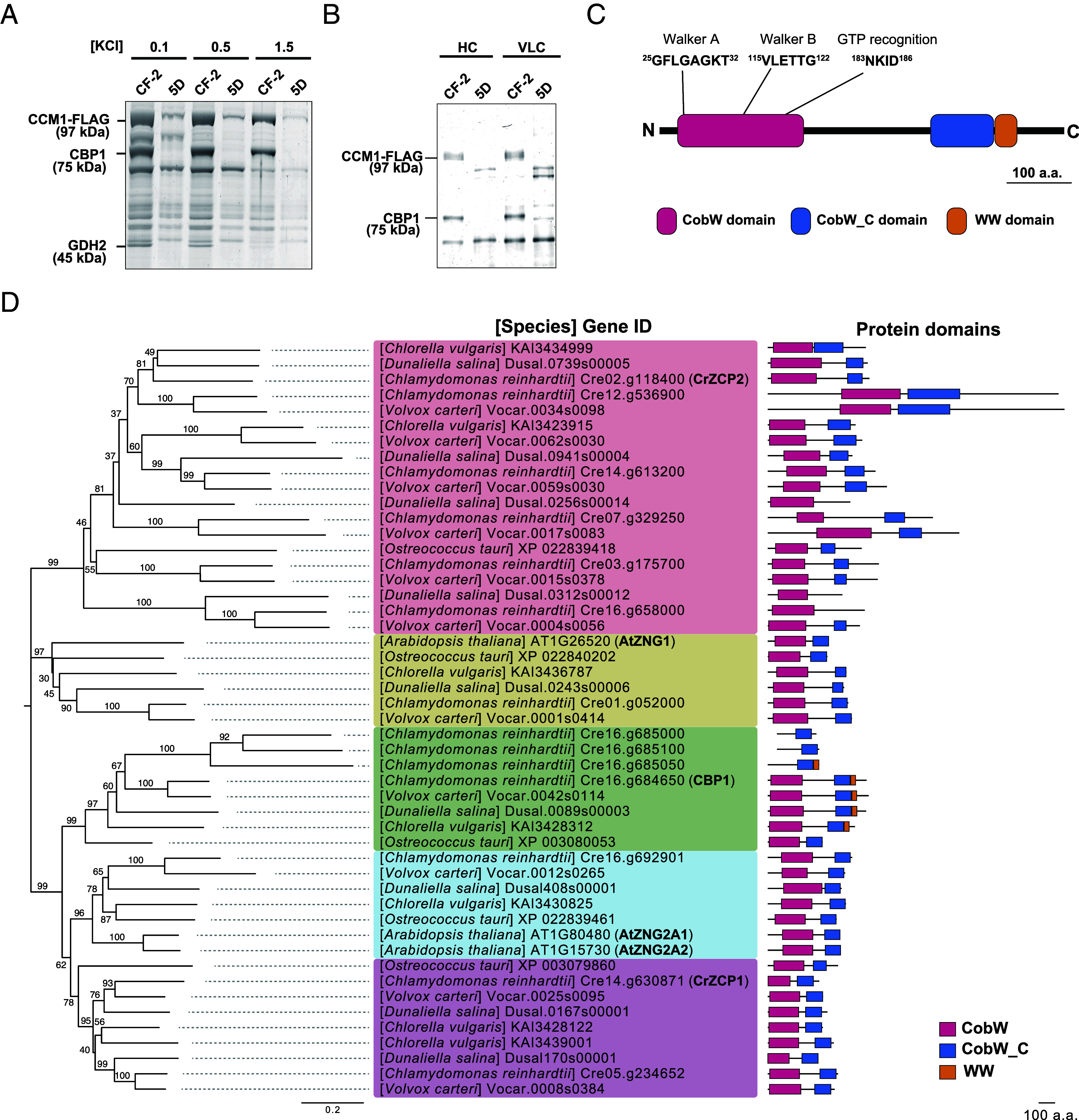
CBP1, a CobW/WW-domain-containing protein, is identified as a CCM1-interacting partner. (*A*) The CCM1-FLAG protein complex was isolated from C16(*ccm1*):*CCM1-FLAG* #2 (labeled as CF-2) by pull-down assay and subjected to SDS-PAGE followed by silver staining. The arrows indicate the positions of the major components of the complex, with their estimated molecular weights. Cells were cultured under heterotrophic conditions. The KCl concentration in the wash buffer is indicated. Note that the 45 kDa band (GDH2) dissociates at 1.5 M KCl, whereas the 75 kDa band (CBP1) remains bound, indicating a tight interaction with CCM1. (*B*) Accumulation of CBP1 in CCM1-FLAG complexes isolated from C16 (*ccm1*):*CCM1-FLAG* #2 (CF-2) cells grown under HC (5% CO_2_) and VLC (0.04% CO_2_) conditions. 5D represents the parental strain used to generate the transformant. CBP1 was detected by immunoblot analysis using anti-CBP1 antibody. CCM1-FLAG was detected using anti-FLAG antibody. (*C*) Domain structure and motif sequence of the CBP1 protein. The CobW domain contains conserved amino acid motifs important for GTPase activity including the P-loop (GxxxxGKT), Switch I (DxxG), and Switch II (NKxD) motifs. (*D*) Phylogenetic tree of COG0523 proteins conserved in *Arabidopsis thaliana*, *C. reinhardtii*, *Volvox carteri*, *Dunaliella salina*, *Chlorella vulgaris,* and *Ostreococcus tauri*. The phylogenetic tree was constructed using the Neighbor-Joining (NJ) method based on the amino acid sequences of CBP1 homologs. Bootstrap values (1,000 replicates) are shown at the nodes. The scale bar of the phylogenetic tree represents 0.2 substitutions per site. Protein domains are illustrated to the right of each sequence: CobW (pink), CobW_C (blue), and WW (orange) domains. CBP1 and ZNG1 orthologs are highlighted in bold.

Pull-down experiments revealed that the CCM1-FLAG complex contained two major components beyond CCM1 itself (Fig. 1*A*). In CF-2 cells, two distinct protein bands of approximately 75 and 45 kDa were visible on SDS-PAGE. LC–MS/MS identified the 75 kDa band as a previously uncharacterized protein encoded by *Cre16.g684650*, while the 45 kDa band corresponded to glutamine dehydrogenase 2 (GDH2) (*SI Appendix*, Fig. S2 and Tables S1 and S2). To assess binding strength, we performed washes with increasing KCl concentrations (0.1, 0.5, and 1.5 M). A 1.5 M KCl wash abolished the GDH2 signal but not the 75 kDa protein, indicating that GDH2 associates only weakly with CCM1, whereas the 75 kDa protein forms a tight complex (Fig. 1*A*). We therefore named the 75 kDa protein as CCM1-binding protein 1 (CBP1). A parallel analysis using in-solution tryptic digestion of the entire CCM1-FLAG eluate corroborated these findings, confirming the presence of CBP1 and GDH2, and additionally identifying glutamate dehydrogenase 1 (GDH1) as another component of the complex (*SI Appendix*, Fig. S2 and Table S3).

Subsequent immunoblot analyses showed that CBP1 is present at similar levels in cells grown under both high-CO_2_ (HC, 5% CO_2_) and very-low-CO_2_ (VLC, 0.04% CO_2_) conditions (Fig. 1*B*). Furthermore, a reciprocal coimmunoprecipitation assay demonstrated that an anti-CBP1 antibody successfully coprecipitated CCM1 under both HC and VLC conditions (*SI Appendix*, Fig. S1*B*). Together, these results suggest that the CCM1–CBP1 interaction occurs constitutively and does not depend on external CO_2_ levels. These findings prompted us to investigate CBP1’s role in CCM regulation under varying CO_2_ conditions. Notably, mass spectrometric analysis revealed that CCM1 undergoes phosphorylation at Ser122 and Ser451 under both HC and VLC conditions, whereas phosphorylation at Ser16 was exclusively detected under VLC conditions (*SI Appendix*, Fig. S2 and Table S4). These experimentally verified modifications suggest additional layers of posttranslational regulation that may modulate CCM1 transcriptional activity in response to CO_2_ availability.

### CBP1 Contains a CobW/CobW_C Domain and a WW Domain.

Sequence analysis revealed that CBP1 possesses three recognizable domains: a CobW domain, a CobW_C domain, and a WW domain (Fig. 1*C*). The CobW domain contains conserved Walker A (residues 25 to 32: GFLGAGKT) and Walker B (residues 115 to 122: VLETTG) motifs, as well as a GTP recognition sequence (residues 183 to 186: NKID), all essential for GTPase activity. Proteins harboring CobW/CobW_C typically belong to the COG0523 family, known for metal chaperone activity (21). In Viridiplantae, two COG0523-type proteins—Zn-regulated GTPase metalloprotein activator 1 (ZNG1) and ZNG2—are broadly conserved (23). In *Chlamydomonas*, ZCP1 and ZCP2 similarly encode COG0523 proteins that are induced under zinc-deficient conditions (21). Unlike these characterized COG0523 proteins, CBP1 also features a C-terminal WW domain, which typically mediates protein–protein interactions through proline-rich sequences.

Phylogenetic analysis of COG0523 proteins across green algae and plants indicates that CBP1 belongs to a separate clade from ZNG1/2 and that WW-domain-containing COG0523 proteins are restricted to certain green algal lineages (Chlorophyceae and Trebouxiophyceae), precisely mirroring CCM1’s taxonomic distribution (Fig. 1*D* and *SI Appendix*, Fig. S3). This co-occurrence strongly suggests coevolution of the CBP1-CCM1 regulatory module. These observations suggest that CBP1 exerts a distinct nuclear function in *Chlamydomonas*, potentially involving metal ion transfer and protein-binding events.

**Fig. 2.**
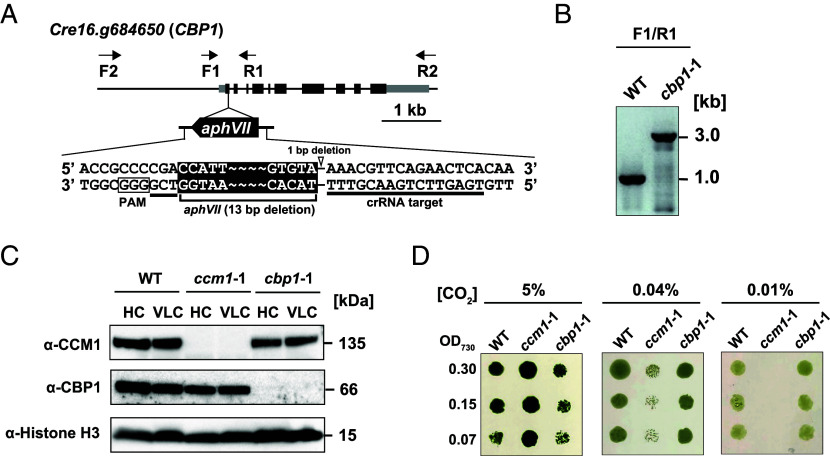
Loss of CBP1 does not affect growth under various CO_2_ conditions. (*A*) Schematic representation of the *aphVII* cassette insertion in the *cbp1* mutant. Exons, introns, and untranslated regions are shown as black boxes, black lines, and gray boxes, respectively. The *aphVII* cassette was inserted into the first exon in the orientation opposite to *CBP1* transcription. The expanded view shows the nucleotide sequences of the crRNA target site (underlined) and the PAM motif (boxed). The insertion event was accompanied by a 1-bp deletion in the *CBP1* genomic sequence (indicated by an open arrowhead) and a 13-bp deletion at the end of the *aphVII* cassette. Binding sites of PCR primers are indicated as arrows: F1 and R1 primers were used for genotyping to confirm *aphVII* cassette insertion; F2 and R2 primers were used to amplify the *CBP1* genomic fragment for complementation. (*B*) Genomic PCR to confirm insertion of the *aphVII* cassette in the *CBP1* gene. (*C*) Accumulation of CCM1 and CBP1 in WT, *ccm1* mutant (*ccm1*-1), and *cbp1* mutant (*cbp1*-1). Cells grown in 5% CO_2_ were transferred to 5% (HC) or 0.04% (VLC) CO_2_ for 12 h. Histone H3 was used as a loading control. (*D*) Spot tests of WT, *ccm1*-1, and *cbp1*-1. Cells grown to logarithmic phase were diluted to the indicated optical density (OD_730_ = 0.30, 0.15, or 0.07), and the cell suspensions were spotted on MOPS-P agar plates. Cells were grown in closed chambers supplied with 5%, 0.04%, or 0.01% CO_2_ under continuous light at 120 μmol photons m^−2^ s^−1^. Images were taken after 3 d of growth.

To test CBP1’s role in CCM regulation, we generated a *cbp1* mutant by CRISPR/Cas9-directed insertion of an *aphVII* resistance cassette into the first exon of *CBP1* in the wild-type strain C9 (Fig. 2*A*). Genomic PCR confirmed the successful insertion of the cassette, with the mutant showing a 3.0 kb band compared to the 1.0 kb wild-type band (Fig. 2*B*).

To examine protein accumulation, we analyzed CCM1 and CBP1 levels under HC and VLC conditions. Immunoblotting with an anti-CBP1 antibody confirmed that the resulting mutant (*cbp1*-1) completely lacked CBP1 protein under both HC and VLC conditions (Fig. 2*C*). Importantly, CCM1 protein levels in the *cbp1*-1 mutant were comparable to those in wild-type under VLC conditions. Likewise, CBP1 protein levels in the *ccm1*-1 mutant (26) were also similar to those in wild-type under both HC and VLC conditions (Fig. 2*C*). These results demonstrate that the accumulation (or stability) of CCM1 and CBP1 does not require their interaction.

Growth analysis revealed striking differences between the *cbp1* and *ccm1* mutants. While *ccm1*-1 cells showed severe growth defects at 0.04% and 0.01% CO_2_, *cbp1*-1 cells grew normally at all CO_2_ concentrations tested (5%, 0.04%, and 0.01%) (Fig. 2*D*). This normal growth phenotype indicates that CBP1, unlike CCM1, is dispensable for CCM induction and cell survival under low CO_2_ conditions. Thus, CBP1 is dispensable for growth under both high and low CO_2_ conditions, implying a function distinct from that of CCM1.

Attempts to create a CRISPR/Cas9-directed *GDH1/2* insertion mutants with four independent guide RNAs failed to yield viable transformants (>1,000 colonies screened), suggesting GDH1/2 is essential under our culture conditions and may relay metabolic information to CCM. This possibility is considered further in *Discussion*.

### CBP1 Interacts with CCM1 in the Nucleus.

**Fig. 3.**
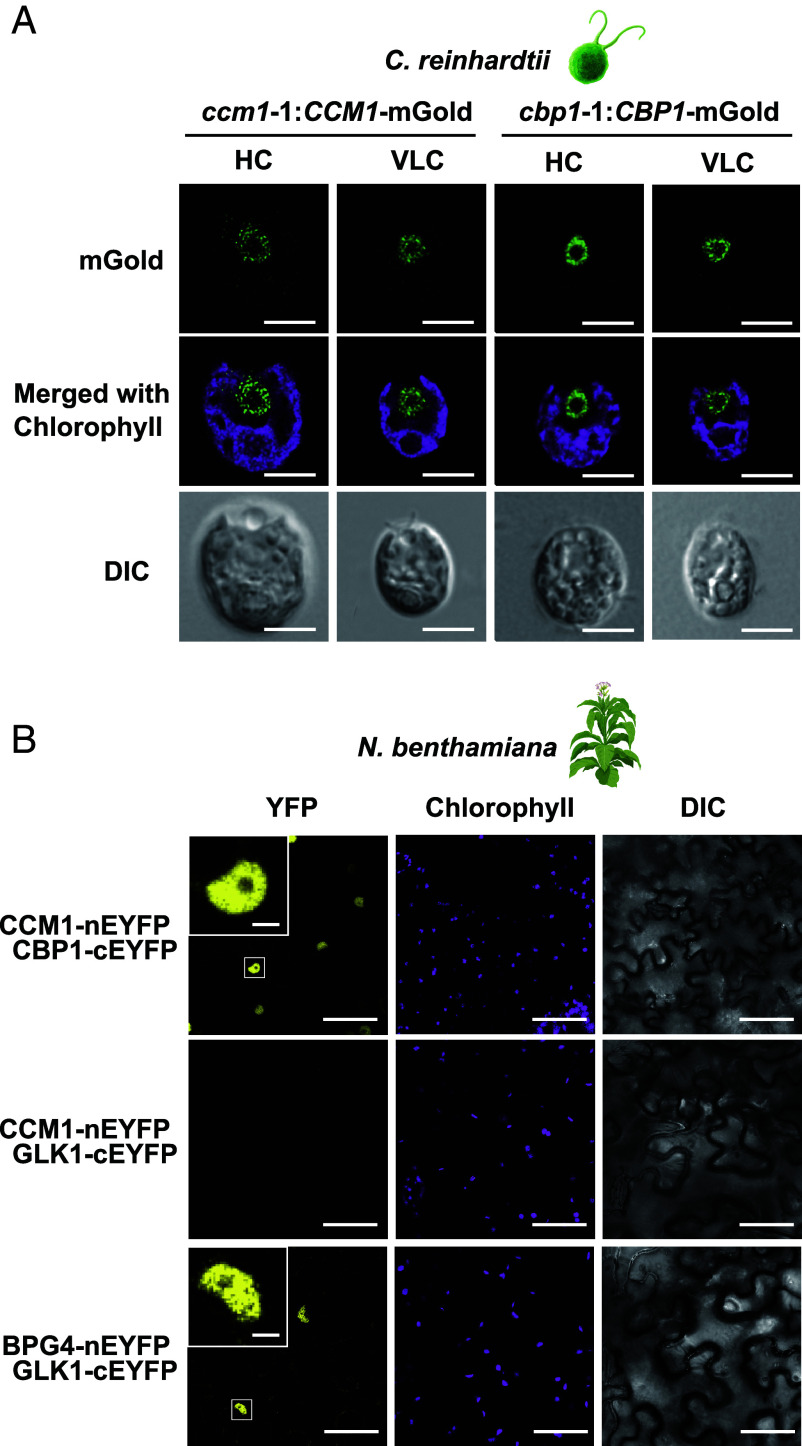
CBP1 localizes to the nucleus and physically interacts with CCM1. (*A*) Intracellular localization of CCM1 and CBP1-mGold fusion proteins in *C. reinhardtii* cells. Cells were observed under conditions acclimated to either 5% (HC) or 0.04% CO_2_ (VLC) for 12 h. Yellow fluorescence indicates the localization of fusion proteins. Chl represents chlorophyll autofluorescence. DIC shows differential interference contrast images. (Scale bars, 5 μm.) (*B*) BiFC assay to evaluate the nuclear interaction between CCM1 and CBP1. CCM1 fused to the N-terminal fragment of EYFP (CCM1-nEYFP) and CBP1 fused to the C-terminal fragment (CBP1-cEYFP) were transiently coexpressed in leaf epidermal cells of *Nicotiana benthamiana*. Reconstituted YFP fluorescence indicates protein–protein interaction. As a negative control, CCM1-nEYFP was coexpressed with the unrelated *Arabidopsis* nuclear protein GLK1-cEYFP (middle row) to verify the absence of nonspecific background fluorescence. The known interaction between Arabidopsis BPG4-nEYFP and GLK1-cEYFP served as a positive control (bottom row). White boxes in the YFP panels indicate regions shown in the enlarged images. Insets show enlarged views of the boxed regions. [Scale bars, 50 μm (main panels) and 5 μm (*Insets*).]

Because CCM1 localizes to the nucleus, we hypothesized that CBP1 would function in the same compartment. To visualize CBP1’s localization, we fused CBP1 to the fluorescent protein mGold and introduced the construct into *cbp1*-1, generating *cbp1*-1:*CBP1*-mGold. In parallel, we fused CCM1 to mGold and introduced it into *ccm1*-1, yielding *ccm1*-1:*CCM1*-mGold. Confocal microscopy revealed strong nuclear signals in both transformants under HC and VLC conditions (Fig. 3*A*). The mGold signals clearly localized to a distinct nuclear region that did not overlap with chlorophyll autofluorescence, as shown in the merged images. Notably, the nuclear localization pattern remained unchanged between HC and VLC conditions for both proteins, showing punctate nuclear signals characteristic of transcription factor localization (Fig. 3*A*).

To confirm i*n vivo* CCM1–CBP1 interactions, we employed bimolecular fluorescence complementation (BiFC), in which YFP is split into nEYFP and cEYFP fragments that fluoresce only when brought together by an interacting protein pair. We tagged CCM1 and CBP1 with nEYFP and cEYFP, respectively, and transiently expressed them in *N. benthamiana* leaves. A bright nuclear YFP signal was observed exclusively when CCM1-nEYFP and CBP1-cEYFP were coexpressed (Fig. 3*B*). Given the lack of established BiFC control pairs from *Chlamydomonas*, we utilized the *Arabidopsis* nuclear proteins GLK1 and BPG4, a well-characterized interacting pair (27), as controls. While the positive control pair (BPG4-nEYFP and GLK1-cEYFP) reconstituted fluorescence, the negative control pairing of CCM1-nEYFP with GLK1-cEYFP yielded no signal. This confirms that the interaction is specific and not due to self-assembly of the split YFP fragments. Together with our pull-down results, these data demonstrate that CCM1 and CBP1 physically interact in the nucleus, regardless of external CO_2_ availability.

### CBP1 Represses Several CCM-Related Genes Under HC Conditions.

To determine whether CBP1 influences CCM gene expression, we performed RNA-seq on strains lacking or complementing both CCM1 and CBP1 under HC and VLC. Specifically, we generated *ccm1*-1:*CCM1* by introducing a functional *CCM1* genomic fragment into *ccm1*-1, selecting a transformant whose Ci-affinity under VLC matched that of WT (*SI Appendix*, Table S5). Likewise, *cbp1*-1:*CBP1* was established by reintroducing the *CBP1* genomic fragment into the *cbp1*-1 mutant background.

**Fig. 4.**
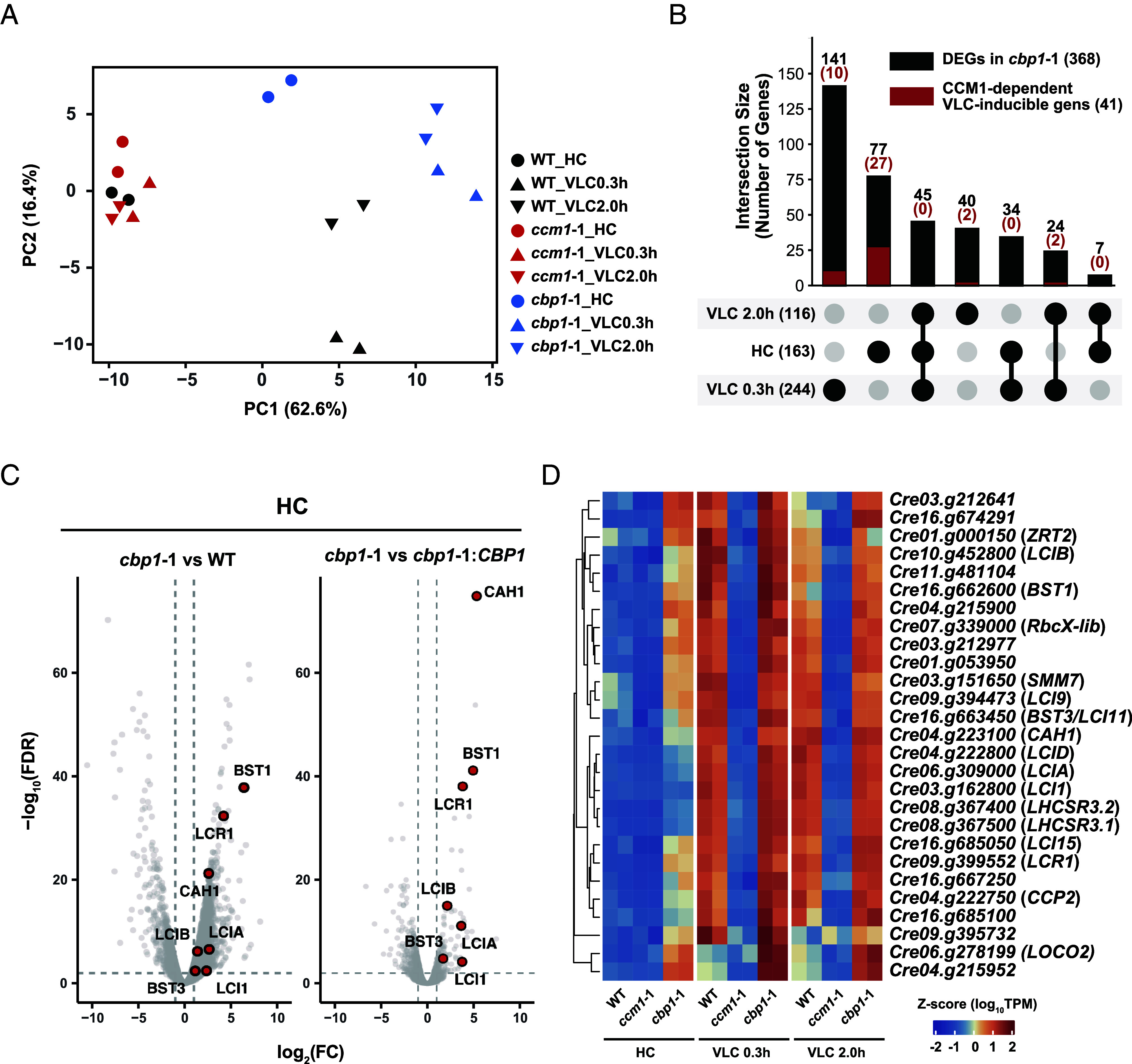
CBP1 represses CCM gene expression under high CO_2_ conditions. (*A*) PCA plot visualizing expression patterns of VLC-inducible genes in each sample. The plot shows the first two principal components (PC1 and PC2), respectively. Samples are color-coded and shape-coded by strain (WT, *ccm1*-1, *cbp1*-1) and CO_2_ condition (HC, VLC 0.3 h, VLC 2.0 h). PC1 accounts for 62.6% of the variance and PC2 for 16.4%. (*B*) UpSet plot illustrating the intersections of differentially expressed genes (DEGs) identified in the *cbp1*-1 mutant across different CO_2_ conditions (HC, VLC 0.3 h, and VLC 2.0 h). The vertical bars in the *Upper* panel indicate the number of DEGs (Intersection Size) belonging to a specific intersection, sorted by size. Black bars represent the total number of CBP1-dependent DEGs, while the overlaid red bars represent the subset of 41 CCM1-dependent, VLC-inducible genes. The red numbers in parentheses indicate the count of this subset within each intersection. The connected black dots in the lower matrix indicate the combination of conditions corresponding to the bar directly above. The total number of DEGs for each condition is shown in parentheses next to the condition label. (*C*) Volcano plots showing DEGs in *cbp1*-1 mutants acclimated to HC conditions. *Left* panel: *cbp1*-1 vs WT; *Right* panel: *cbp1*-1 vs *cbp1*-1:*CBP1*. The x-axis shows log_2_ fold change, and the y-axis shows –log_10_(FDR). Red dots indicate Ci transporters, carbonic anhydrases, and the transcription factor involved in CCM which are highlighted and labeled. Dashed lines indicate the threshold for significance (FDR < 0.01, |log_2_FC| > 1). (*D*) Heatmap showing the expression patterns of 27 VLC-inducible and CCM1-dependent DEGs that are misregulated in HC condition across different strains (WT, *ccm1*-1, *cbp1*-1) and CO_2_ conditions (HC, VLC 0.3 h, VLC 2.0 h). Gene expression levels are represented as Z-scores of log_10_(TPM) values. Gene IDs and their corresponding gene names are shown on the right. Hierarchical clustering was performed on both genes (rows) and conditions (columns).

We then cultured WT, *ccm1*-1, *cbp1*-1, *ccm1*-1:*CCM1*, and cbp1-1:*CBP1* under HC for 24 h, shifted them to VLC for 0.3 or 2.0 h, and analyzed their transcriptomes using Trimmed Mean of M-values (TMM) normalization. We first defined VLC-inducible genes as those significantly upregulated (FDR < 0.01, log_2_ fold change > 1) after 0.3 or 2.0 h under VLC in WT. A principal component analysis (PCA) of these VLC-inducible genes revealed that *cbp1*-1 displayed a distinct expression profile compared to WT or *ccm1*-1, with HC samples clustering separately from VLC samples along PC1 (62.6% variance), while *cbp1*-1 samples under HC diverged from other HC samples along PC2 (16.4% variance), pointing to a unique role for CBP1 (Fig. 4*A*).

Next, we classified DEGs as CCM1-dependent or CBP1-dependent if their expression in the mutant (either *ccm1*-1 or *cbp1*-1) differed significantly from both WT and the respective complemented strain under any condition (FDR < 0.01, |log_2_ fold change| > 1). Of the 368 CBP1-dependent DEGs identified, 163 were affected under HC conditions, while 244 and 116 showed altered expression at VLC 0.3 h and 2.0 h, respectively (Fig. 4*B*). We mapped the 41 CCM1-dependent VLC-inducible genes onto this dataset (indicated by red bars in Fig. 4*B*). Notably, the majority of these CCM1-dependent genes (27 out of 41) overlapped specifically with the set of genes derepressed in *cbp1*-1 under HC conditions. This indicates that while CBP1-dependent DEGs are distributed across all conditions, CBP1 is specifically required to repress the HC-inappropriate expression of this CCM1-dependent subset.

Given that *cbp1*-1 exhibited a unique transcriptional profile under HC (Fig. 4*A*), we examined HC-specific gene expression changes in detail. Volcano plot analysis revealed substantial derepression of key CCM components, including carbonic anhydrases (CAH1, LCIB), Ci transporters/channels (LCIA, LCI1, BST1, BST3), and transcription factor LCR1 (Fig. 4 *C*, *Left* panel and *SI Appendix*, Table S6). The significant upregulation of *CAH1* is likely a, downstream consequence of the strong induction of *LCR1*, a Myb transcription factor known to directly activate *CAH1* expression (7). Complementation analysis (*cbp1*-1 vs. *cbp1*-1:*CBP1*) confirmed that reintroduction of *CBP1* restored repression of these CCM genes under HC conditions, validating that the observed derepression is specifically due to CBP1 loss. It is also of note that *CAH1* showed enhanced suppression in the complemented strain (Fig. 4 *C*, *Right* panel).

Among VLC-inducible, CCM1-dependent genes, 27 genes were derepressed in *cbp1*-1 specifically under HC (Fig. 4*B* and *SI Appendix*, Table S6). Hierarchical clustering of the 27 misregulated genes revealed coordinated upregulation in *cbp1*-1 under HC, with Z-scores ranging from 0 to 2, indicating substantial derepression (Fig. 4*D*). We also observed upregulation of light stress-related genes (*LHCSR3.1* and *LHCSR3.2*) and a mitochondria-localized membrane protein (*CCP2*) in *cbp1*-1 under HC. The heatmap demonstrates that these genes maintained normal VLC-induced expression in *cbp1*-1, suggesting CBP1 specifically functions in HC repression rather than VLC activation (Fig. 4*D* and *SI Appendix*, Table S6). Under VLC, however, the expression of these genes in *cbp1*-1 closely resembled WT. We also did detect altered expression of several unknown genes in *cbp1*-1 under VLC (*SI Appendix*, Table S7), suggesting additional functions for CBP1. Collectively, these results indicate that CBP1 acts primarily as a repressor of CCM-related genes under HC conditions.

### Inorganic Carbon Affinity Is Increased in *cbp1* Mutants Acclimated to HC Conditions.

**Fig. 5.**
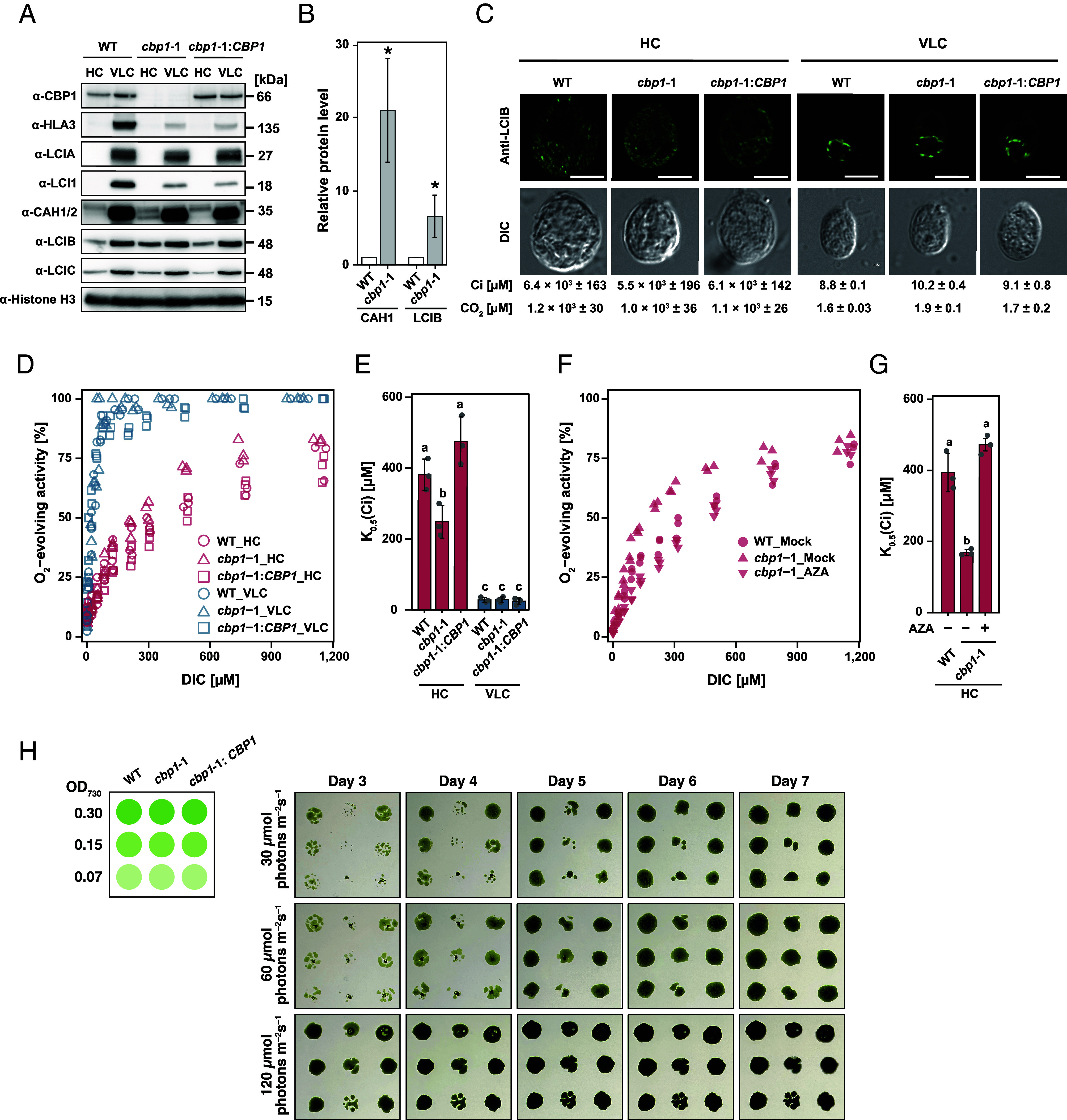
CBP1 loss enhances apparent CO_2_ affinity through elevated carbonic anhydrase activity. (*A*) Accumulation of CCM-related proteins in WT, *cbp1*-1, and *cbp1*-1:*CBP1*. Cells were first grown under 5% (v/v) CO_2_ condition for 24 h and shifted to 5% (v/v) CO_2_ (HC) or 0.04% (v/v) CO_2_ (VLC) conditions for 12 h. Western blot analysis was performed using antibodies against the indicated proteins. Identification of CAH1 and CAH2 bands was performed as described previously (28). Histone H3 was used as a loading control. (*B*) Relative protein levels of CAH1 and LCIB of WT and *cbp1*-1 under HC condition were determined. Band intensities were quantified by ImageJ and normalized to Histone H3 as a loading control. Values are expressed relative to WT. Data represent means ± SE from four independent biological replicates. Statistical significance was assessed using Student’s *t* test, with asterisks indicating statistically significant differences (*P < 0.05) compared to WT. (*C*) Subcellular localization of LCIB in WT, *cbp1*-1, and *cbp1*-1:*CBP1* strains acclimated to HC and VLC conditions. Anti-LCIB immunofluorescence (green) and DIC images are shown. The concentration of inorganic carbon (Ci) in the culture medium and the calculated CO_2_ concentration are shown. (Scale bars, 5 μm.) (*D*) Net O_2_-evolving activities of WT, *cbp1*-1, and *cbp1*-1:*CBP1* cells grown in HC or VLC conditions for 12 h measured against dissolved inorganic carbon (DIC) concentrations at pH 7.8. Each data point represents an individual measurement from three biological replicates. (*E*) K_0.5_(Ci) values representing the DIC concentration required for half-maximal O_2_ evolution in WT, *cbp1*-1, and *cbp1*-1:*CBP1* cells grown in HC or VLC conditions for 12 h. Data represent mean values ± SE from three biological replicates. Statistical analysis was conducted using the Tukey–Kramer multiple comparison test, with different letters indicating significant differences (*P* < 0.05). (*F*) Net O_2_-evolving activities of WT and *cbp1*-1 cells treated with or without acetazolamide (AZA) grown in HC conditions for 12 h measured against DIC at pH 7.8. Mock treatment with 1% DMSO was used as vehicle control. (*G*) K_0.5_(Ci) values of WT and *cbp1*-1 treated with or without AZA. AZA was adjusted to a concentration of 5 mM, dissolved in DMSO, and added to the measuring buffer at 1% (v/v). Data represent mean values ± SE from three biological replicates. Statistical analysis was performed using the Tukey–Kramer multiple comparison test, with different letters indicating statistically significant differences (*P* < 0.05). (*H*) Time-course analysis of growth phenotypes under different light intensities with 5% CO_2_. Cells of WT, *cbp1*-1, and *cbp1*-1:*CBP1* grown to the logarithmic phase were diluted to the indicated optical densities (OD_730_ = 0.30, 0.15, and 0.07). Cell suspensions were spotted onto MOPS-P agar plates as depicted in the schematic layout (*Left*). Cells were grown under continuous light at the indicated intensities (30, 60, or 120 μmol photons m^–2^s^–1^) with a constant supply of 5% CO_2_. Images were taken daily from Day 3 to Day 7 after spotting.

To investigate whether the transcriptional derepression observed in *cbp1*-1 leads to functional consequences, we examined the accumulation of key CCM proteins. Immunoblot analysis revealed that under HC conditions, *cbp1*-1 showed increased accumulation of CAH1 and LCIB proteins compared to WT (Fig. 5*A*). Although the α-CAH1 antibody cross-reacts with the HC-inducible CAH2 isoform (upper band, 38 kDa), the CAH1-specific signal (lower band, 36 kDa) was clearly elevated in the mutant. Quantitative analysis with four biological replicates confirmed that CAH1 and LCIB levels were significantly increased by approximately 20-fold and 7-fold, respectively, in *cbp1*-1 under HC conditions (Fig. 5*B* and *SI Appendix*, Fig. S4). This elevated protein accumulation was reversed in the complemented strain (*cbp1*-1:*CBP1*), confirming that CBP1 directly regulates these CCM components. Other CCM proteins, including LCIA, LCI1, LCIC, and HLA3, showed similar levels across all strains under HC conditions. Under VLC, all strains exhibited robust induction of CCM proteins, with *cbp1*-1 showing slightly reduced levels of HLA3 and LCI1, though this was not restored by complementation and thus appears CBP1-independent.

We next examined LCIB localization, as its redistribution from diffuse to punctate pattern around pyrenoid is a hallmark of CCM activation. Despite the increased LCIB protein levels in *cbp1*-1 under HC, its localization remained diffuse in all strains under HC conditions (Ci: 5.5 to 6.4 × 10^3^ µM, CO_2_: 1.0 to 1.2 × 10^3^ µM), similar to WT (Fig. 5*C*). Under VLC conditions (Ci: 8.8 to 10.2 µM, CO_2_: 1.6 to 1.9 µM), LCIB formed distinct puncta in all strains, indicating that CBP1 loss does not affect LCIB relocalization dynamics. To assess whether increased CAH1 and LCIB levels enhance CO_2_ utilization, we measured photosynthetic O_2_ evolution across a range of DIC concentrations. The *cbp1*-1 mutant acclimated to HC showed enhanced apparent Ci affinity compared to WT (Fig. 5 *D* and *E*). Quantification revealed that K_0.5_(Ci), the DIC concentration supporting half of maximal O_2_ evolution (V_max_), decreased from 381 ± 26 µM in WT to 248 ± 27 µM in *cbp1*-1 under HC conditions (*P* < 0.05), while complementation restored it to 475 ± 40 µM (Fig. 5*D* and *SI Appendix*, Table S5). Under VLC conditions, all strains showed similarly low K_0.5_(Ci) values (~20 to 30 µM), confirming that the enhanced affinity phenotype is HC-specific. V_max_ were comparable across all strains under both conditions (*SI Appendix*, Table S5).

To determine whether the enhanced Ci affinity in *cbp1*-1 results from elevated CA activity, we treated cells with acetazolamide (AZA), a membrane-impermeable inhibitor of extracellular CAs including CAH1. AZA treatment (5 mM) increased K_0.5_(Ci) in HC-acclimated *cbp1*-1 from 169 ± 5 µM to 416 ± 70 µM, effectively restoring it to WT levels (394 ± 31 µM) (Fig. 5 *F* and *G* and *SI Appendix*, Table S5). Similarly, we previously demonstrated that AZA treatment of WT cells under VLC conditions increases K_0.5_(Ci) from ~25 μM to ~150 to 200 μM (28), establishing the essential role of extracellular carbonic anhydrase activity in high-affinity Ci uptake. This comparison validates that the modest increase in CAH1 protein levels observed in *cbp1*-1 (Fig. 5 *A* and *B*) is physiologically sufficient to significantly enhance Ci affinity. Thus, the complete rescue by AZA confirms that the enhanced Ci affinity in *cbp1-1* is strictly dependent on excess extracellular CA activity, which likely operates in concert with upregulated intracellular components such as LCIB.

Given that *cbp1*-1 exhibits enhanced Ci affinity due to unnecessary CCM activation, we hypothesized that this deregulation might impose a metabolic cost affecting growth. In our initial spot tests (Fig. 2*D*), while *cbp1*-1 clearly lacked the severe growth defects characteristic of *ccm1* mutants under 0.04% and 0.01% CO_2_, it appeared to exhibit a subtle growth difference compared to WT under 5% CO_2_. However, the strong saturation of growth in those standard assays made it difficult to determine whether this was a genuine physiological defect. To rigorously assess the energetic burden of CCM derepression, we therefore performed a detailed time-course growth analysis under limiting light intensities, where photosynthetic ATP supply would be restricted. As shown in Fig. 5*H*, under standard light (120 μmol photons m^–2^ s^–1^), *cbp1*-1 exhibited a slight transient growth delay compared to WT during the early phase (Day 3 to 4), which became negligible by Day 6 to 7 as growth saturated. In contrast, under limiting light (30 to 60 μmol photons m^–2^ s^–1^), *cbp1*-1 showed a significant and persistent growth defect compared to WT and the complemented strain. This demonstrates that the inappropriate accumulation of CCM proteins imposes a fitness cost that is exacerbated when photosynthetic ATP production is limited.

Collectively, these results reveal that CBP1 maintains appropriate CCM suppression under HC conditions by preventing premature accumulation of CCM proteins, particularly CAH1. Loss of this regulatory control leads to constitutive CA activity that enhances CO_2_ capture efficiency even under carbon-replete conditions, potentially representing an energetic burden to the cell.

## Discussion

**Fig. 6.**
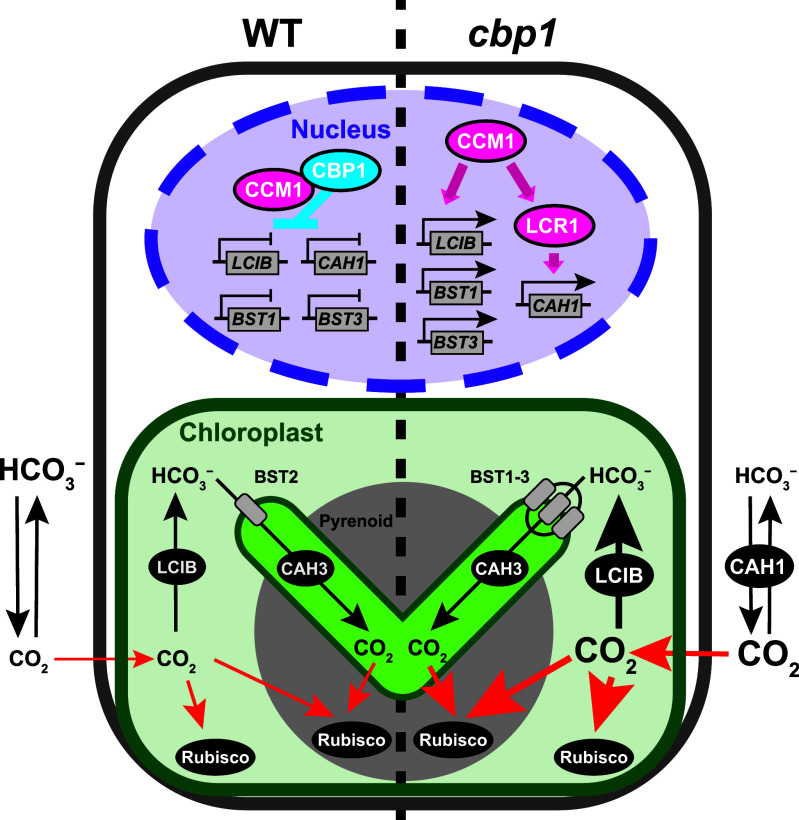
Model illustrating the regulatory role of CBP1 in suppressing CCM gene expression under high CO_2_ conditions. Under HC conditions, the model compares CCM regulation between wild-type (WT, *Left*) and *cbp1* mutant (*Right*) cells. In WT cells, CBP1 physically interacts with CCM1 in the nucleus, preventing CCM1 from activating transcription of CCM genes including *BST1*, *BST3*, *LCIB*, and *CAH1* (indicated by repression bars on gene promoters). This results in minimal expression of CCM components, with only basal levels of carbonic anhydrases (LCIB in chloroplast stroma and CAH3 in thylakoid membranes). CO_2_ passively diffuses across membranes (red arrows) to reach Rubisco. In *cbp1* mutant cells (*Right*), the absence of CBP1-mediated repression allows CCM1 to initiate a complex transcriptional cascade even under HC conditions. This cascade includes the activation of the transcription factor LCR1, which in turn induces its direct target, *CAH1*. In parallel, other key CCM genes such as *BST1*, *BST3*, and *LCIB* are also activated (shown by active transcription from gene promoters), although whether this is due to direct regulation by CCM1 or mediated by other unidentified transcription factors remains unknown. This widespread derepression leads to the increased accumulation of CAH1 and LCIB proteins, as confirmed by immunoblot analysis (Fig. 5*A*). The elevated CAH1 in the periplasmic space enhances the dehydration of HCO_3_^−^ to CO_2_. This increased carbonic anhydrase activity enhances the local CO_2_ concentration at the cell surface, improving CO_2_ capture efficiency and thereby explaining the enhanced apparent Ci affinity observed in *cbp1* mutants. The BST1-3 proteins, which function as HCO_3_^–^ transporters, also show increased expression.

In this study, we identified a nuclear repressor that suppresses the algal CCM when CO_2_ becomes abundant. By screening for CCM1 interactors, we found CBP1, a CobW/WW-domain protein that localizes to the nucleus, binds CCM1 constitutively, and is required to repress a defined subset of CCM1-dependent genes under HC conditions (Fig. 6). This finding addresses a significant gap in our understanding of how green algae deactivate the CCM and establishes a Zn-linked regulatory layer that complements the LysR-type and bZIP-type repressors known in cyanobacteria and diatoms.

### Zn-Linked Control of CCM1 Activity.

CBP1 belongs to the GTP-driven COG0523 family, members of which are involved in divalent metal trafficking (20–22). This structural feature is intriguing given that CCM1 requires two tightly bound Zn^2+^ ions for its structural integrity and transcriptional activity (13, 14). Based on these observations, we hypothesize that CBP1 may function as a metallochaperone that modulates the Zn^2+^ occupancy or metal status of CCM1, thereby regulating its activity. While our BiFC and pull-down assays confirm a physical interaction between CBP1 and CCM1, demonstrating direct zinc transfer and its functional consequences remains an essential objective for future biochemical studies. The finding of CBP1 provides a clear molecular candidate for linking intracellular metal availability to CCM gene regulation.

Although the transcriptional derepression of CAH1 and other CCM genes in *cbp1* mutants under HC is partial compared to their full induction under VLC, it results in sufficient protein accumulation to alter the physiological Ci affinity. This suggests that while CBP1 is a key component of HC repression, other factors, potentially including the identified CBP1 paralogs, likely cooperate to achieve the complete transcriptional silencing observed in wild-type cells.

### CBP1-Dependent Genes and Potential Regulatory Mechanisms.

Loss of CBP1 triggers the derepression of a broad set of CCM genes under HC conditions. Notably, the upregulated genes in *cbp1* mutants, including LCI1, BST1, BST3, LCIB, and CAH1, correspond closely to the components of the “low-affinity CCM” (L-CCM) mode (29). Physiologically, the L-CCM mode facilitates CO_2_ uptake and trapping: entering CO_2_ is converted to HCO_3_^–^ by the stromal LCIB/LCIC complex and subsequently imported into the thylakoid lumen by BST1/3. This contrasts with the “high-affinity CCM” (VLC) mode, which relies on active HCO_3_^–^ uptake driven by HLA3 at the plasma membrane and LCIA at the chloroplast envelope. This functional distinction is strongly supported by our protein-level analysis; while the L-CCM components CAH1 and LCIB significantly accumulate in *cbp1* mutants under HC (Fig. 5 *A* and *B*), the VLC components HLA3 and LCIA do not show comparable accumulation (Fig. 5*A*). This suggests that CBP1 functions primarily as a global repressor of the L-CCM program when CO_2_ is abundant. Furthermore, the observation that VLC-type uptake system remains suppressed at the protein level implies that the high-affinity CCM mode is subject to distinct regulatory controls. We propose that additional, CBP1-independent repressors likely exist to strictly silence the VLC pathway under carbon-replete conditions, ensuring that the cell does not deploy the most energy-intensive machinery until absolutely necessary.

Our data show that CBP1 also represses several other previously unrecognized genes, suggesting their involvement as CCM components or modulators. For instance, LCI9 and the methyltransferase SMM7 may have roles in pyrenoid assembly or maintenance (30). More importantly, two additional transcription factor genes, *Cre03.g212977* and *Cre03.g212641*, are also derepressed in the *cbp1*-1 mutant (*SI Appendix*, Table S6). These factors, which respond to both low CO_2_ and cold stress (31), are strong candidates for the missing activators that control *LCIB, BSTs*, and other CCM genes in parallel to the CAH1–LCR1–CAH1 axis, potentially via chromatin remodeling (32). The finding of this complex network, governed by a protein with a GTP-dependent zinc transferase domain like CBP1, is intriguing. Functional precedents in yeast and vertebrates link Zn trafficking directly to transcriptional regulation (33, 34), raising the possibility that CBP1 modulates this network via zinc-dependent control of transcription factor activities or chromatin structure. Therefore, further characterization of these CBP1-regulated pathways will be required to understand how zinc metabolism, transcriptional regulation, and stress adaptation are integrated to finely regulate CCM activity.

### A Putative Link between Carbon–Nitrogen Status and CCM Control.

Besides CBP1, our pull-down consistently retrieved glutamate dehydrogenase GDH1/2 (Fig. 1*A* and *SI Appendix*, Tables S1–S4), an enzyme that reversibly converts glutamate and α-ketoglutarate while shuttling NAD(P)H (35). GDH1/2 was lost after 1.5 M KCl washes (Fig. 1*A*) and, unlike CBP1, we failed to isolate GDH1/2 mutants despite four independent CRISPR guides and screening > 1,000 colonies, echoing earlier biochemical work showing that all three NAD(P)^+^-dependent GDH isozymes are mitochondrial and indispensable for amino-acid catabolism in *Chlamydomonas* (36). We speculate that GDH1/2 docks only transiently on CCM1 to couple cellular C/N or redox status to CCM shutdown: When external CO_2_ is high, a rising glutamate:α-ketoglutarate ratio would favor nitrogen assimilation and could provide an additional signal to disengage the CCM. Determining whether GDH1/2 alters CCM1 posttranslationally, or whether its metabolic products modulate the CBP1 Zn-switch, will require conditional GDH knock-down and targeted metabolomics, but the weak yet reproducible interaction uncovered here points to a promising carbon–nitrogen crosstalk layer for future study.

### Physiological Impact, Evolutionary Footprint, and Applied Outlook.

Time-course growth analysis under varying light intensities revealed that the energetic burden of partial CCM derepression in *cbp1*-1 is strictly light-dependent (Fig. 5*H*). Under standard light conditions, *cbp1*-1 exhibits normal growth (or only a minor transient delay), indicating that abundant photosynthetic energy allows cells to largely compensate for the metabolic cost of unnecessary CCM protein expression and operation. However, under light-limiting conditions, this compensation fails, resulting in significant and persistent growth retardation. This confirms that the maintenance and operation of the CCM constitute a substantial energy sink, consistent with recent bioenergetic models (18–20). The fact that *cbp1* mutants exhibit a clear fitness penalty specifically when energy is scarce demonstrates that CBP1 plays a critical role in energy conservation, ensuring that resources are not wasted on the CCM when CO_2_ is abundant. The existence of potential functional redundancy among CBP1 paralogs (Fig. 1*D*) might further contribute to this regulatory robustness, potentially mitigating the severity of the phenotype.

In contrast, the *ccm1*-1 mutant, which is completely unable to induce the CCM, consistently exhibits slightly better growth than wild-type under HC conditions (Fig. 2*D*). This suggests that wild-type cells incur a fitness cost from maintaining even a basal level of the CCM machinery. Indeed, previous transcriptome analyses have established that many CCM component genes maintain a low but distinct basal level of expression in wild-type cells even under HC conditions (11). The complete absence of this basal expression in *ccm1*-1 likely provides a net energy saving that promotes growth when CO_2_ is not limiting. Taken together, the contrasting phenotypes of *cbp1*-1 and *ccm1*-1 highlight the delicate energetic trade-offs associated with CCM regulation and the importance of its strict suppression when CO_2_ is abundant.

The co-occurrence of WW-domain COG0523 proteins and CCM1 exclusively in Chlorophyceae and Trebouxiophyceae suggests that the CCM1–CBP1 module is a lineage-specific solution for reversible CCM control, distinct from the LysR- and bZIP-type repressors used by cyanobacteria and diatoms (15–17). Whether higher plants employ a comparable Zn-based switch for their CO_2_-responsive regulators remains an open and exciting question (2). From an applied perspective, modulating CBP1 activity offers a strategy for metabolic engineering: Attenuating CBP1 could enhance CO_2_ capture from flue gas, while enhancing it might conserve ATP in carbon-rich photobioreactors, provided that zinc homeostasis is maintained. By positioning CBP1 at the nexus of metal metabolism and transcriptional repression, this study offers both a mechanistic framework for nuclear metal signaling and a blueprint for engineering algal CO_2_ fixation.

## Materials and Methods

### *C. reinhardtii* Strains and Culture Conditions.

*C. reinhardtii* strains used were wild-type C9, the *ccm1* mutant (26), and strain 5D (CC-2677). Cells were cultured in Tris-acetate-phosphate (TAP) medium at 25 °C under continuous light (~120 μmol photons m^–2^ s^–1^) and transferred to MOPS-buffered phosphate (MOPS-P) medium for experiments. Cultures were aerated with 5% CO_2_ (HC) or ambient air containing 0.04% CO_2_ (VLC). The VLC condition maintains dissolved CO_2_ <7 μM, corresponding to the physiological state defined by LCIB localization (37).

### Pull-Down Assay.

Cells cultured in MOPS-P medium were harvested and disrupted by sonication in the presence of protease inhibitors. Clarified lysates were incubated with M2 FLAG Affinity Gel (Sigma-Aldrich). To distinguish specific interactors, beads were washed with buffers of increasing ionic strength (100, 500, and 1,500 mM KCl). Bound proteins were eluted with 3× FLAG peptide and analyzed by SDS-PAGE and LC–MS/MS.

### Mass Spectrometry analysis.

Protein bands were excised under blue light using a Safe Imager Blue-Light transilluminator (Invitrogen). Gels previously visualized by FLA3000 were placed on the illuminator, and regions of interest were excised with a surgical blade. Mass spectra were obtained from the gel pieces excised and subsequently digested with trypsin using an LC–MS/MS spectrometer (LTQ: Thermo Fisher Scientific) (38). Protein identification was performed by peptide mass fingerprinting using BioWorks software (Thermo Science).

### Coimmunoprecipitation with Anti-CBP1 Antibody.

Cells were disrupted in PBS containing 0.1% Triton X-100 and protease/phosphatase inhibitors. Clarified lysates were incubated with Dynabeads Protein A (Invitrogen) precoupled with anti-CBP1 antibody. After washing with PBS, bound proteins were eluted with 50 mM glycine (pH 2.8) and analyzed by immunoblotting using anti-CBP1 and anti-CCM1 antibodies.

### Phylogenetic Analysis.

For phylogenetic analysis of COG0523 family proteins, amino acid sequences were retrieved from public databases (NCBI and Phytozome) for *A. thaliana*, *C. reinhardtii*, *V. carteri*, *D. salina*, *C. vulgaris*, and *O. tauri*. Multiple sequence alignment was performed using MAFFT version 7 online service (39). The phylogenetic tree was constructed using the Neighbor-Joining method in MEGA X (40) with 1,000 bootstrap replicates. Domain structures (CobW, CobW_C, and WW domains) were predicted using the NCBI Conserved Domain Database.

### Generation of Anti-CBP1 Antibody.

Polyclonal antibodies were raised in rabbits using purified, full-length recombinant CBP1 expressed in *Escherichia coli* as the antigen. The N-terminal GST tag was removed prior to immunization. The resulting antiserum was affinity-purified using the recombinant CBP1 protein.

### BiFC Assay.

CCM1 and CBP1 were fused to split EYFP fragments (nEYFP and cEYFP). *Arabidopsis* GLK1 and BPG4 served as negative controls (27). The constructs were transiently expressed in *N. benthamiana* leaves via *Agrobacterium*-mediated infiltration as described previously (41) and observed by confocal microscopy.

### Subcellular Localization of CCM1 and CBP1.

Genomic fragments of *CCM1* and *CBP1* were fused with mGold and introduced into the respective mutant backgrounds by electroporation (42). Localization in cells acclimated to HC or VLC conditions was observed using a confocal laser scanning microscope.

### Measurement of Photosynthetic O_2_-Evolving Activity.

Oxygen evolution rates were measured using a Clark-type electrode under saturating light as described previously (43). Cells were resuspended in Ci-depleted HEPES buffer, and NaHCO_3_ was added stepwise. K_0.5_(Ci) values were calculated by fitting data to the Michaelis–Menten equation. For inhibition assays, acetazolamide (AZA) dissolved in DMSO was added to cell suspensions.

### CRISPR-Cas9 System–Mediated Generation of Mutants.

The *cbp1* mutant (*cbp1*-1) was generated using Cas9-gRNA ribonucleoprotein (RNP) complexes targeting the second exon of *CBP1*. The guide RNA was designed using CRISPOR (44). The RNP complex and the *AphVII* cassette (conferring hygromycin resistance) were cointroduced into WT cells by electroporation (45). Transformants were selected with hygromycin, and the disruption of CBP1 was verified by genomic PCR and immunoblot analysis.

### Immunoblot Analysis.

Total protein extracts were prepared in SDS loading buffer, separated by SDS-PAGE, and transferred to PVDF membranes. Immunoblotting was performed using specific primary antibodies against HLA3 and LCIA (46), LCI1 (47), LCIB (48), CAH1/2 (49), and CCM1 (13), as well as anti-CBP1, anti-CAH3 (Agrisera), and anti-Histone H3 (Abcam).

### Generation of Complemented Strains.

To complement *cbp1-1*, a genomic *CBP1* fragment including its promoter was cotransformed with the *AphVIII* marker; restoration of CBP1 was confirmed by immunoblotting. For *ccm1*-1, a genomic fragment from pKI4XA (8) was introduced. Transformants were selected based on photoautotrophic growth under 0.01% CO_2_, and physiological recovery was verified (*SI Appendix*, Table S5).

### RNA-Seq Analysis.

Total RNA was isolated using the RNeasy Plant Mini Kit (QIAGEN) and sequenced on an Illumina NovaSeq 6000 platform with biological duplicates for each condition. Sequence reads were mapped to the *C. reinhardtii* genome (v5.6, Phytozome). The alignment, read counting, and Trimmed Mean of M-values (TMM) normalization of read counts were performed according to the methods previously described (28).

Detailed procedures for all experiments described above are provided in *SI Appendix*.

## Supplementary Material

Appendix 01 (PDF)

## Data Availability

The raw RNA-seq data generated in this study have been deposited in the DDBJ Sequence Read Archive under BioProject accession PRJDB17792 (https://ddbj.nig.ac.jp/search/entry/bioproject/PRJDB17792) (50), with Run accession numbers DRR635765–DRR635812. Strains generated in this study will be available from the Chlamydomonas Resource Center (https://www.chlamycollection.org/) (51) upon publication. The accession numbers of the Phytozome database for *Chlamydomonas* gene *CBP1* is *Cre16.g684650* (52). All other data are included in the manuscript and/or *SI Appendix*.
