# Supplementary material for: A nuclear CobW/WW-domain factor represses the CO2-concentrating mechanism in the green alga Chlamydomonas reinhardtii
Source: Proc Natl Acad Sci U S A. 2026 Feb 4;123(6):e2518136123. doi: 10.1073/pnas.2518136123 (PMC12891040; doi:10.1073/pnas.2518136123)
Supplement: Supplementary file 1 — Appendix 01 (PDF) [file pnas.2518136123.sapp.pdf]

Supporting Information

Materials and methods

Chlamydomonas reinhardtii strains and culture conditions

We used three *Chlamydomonas reinhardtii* strains in this study: (i) a wild-type strain designated C9 (originally from the IAM Culture Collection, University of Tokyo; currently maintained as NIES-2235 and CC-5098), (ii) the *ccm1* mutant (1) , and (iii) strain 5D (CC-2677), a cell-wall-deficient mutant carrying the *nit1* mutation that was used as the parental strain for CCM1-FLAG transformations. We confirmed that this strain exhibits normal CCM induction responses comparable to the wild-type strain C9. Unless otherwise noted, cells were grown in Tris-acetate-phosphate (TAP) medium at 25°C under continuous illumination (~120 μmol photons m^{-2} s^{-1}) with gentle shaking (120 rpm). Once cultures reached mid-logarithmic phase (optical density at 730 nm [OD_{730}] = 0.4–0.7), cells were harvested by centrifugation (2,000 g, 5 min) and transferred to MOPS-buffered phosphate (MOPS-P) medium for experimental treatments.

For high-CO_2 (HC) conditions, cultures were bubbled continuously with 5% (v/v) CO_2 in air, whereas for very low-CO_2 (VLC) conditions, cultures were aerated with ambient air (~0.04% CO_2). This condition maintains the dissolved CO_2 concentration below ~7 μM, corresponding to the physiological VLC state defined by the pyrenoid accumulation of LCIB (37). Cell density was strictly maintained within the mid-logarithmic phase (OD_{730} = 0.3–0.6) to prevent self-shading or gas limitation. Dissolved inorganic carbon (Ci) concentrations in culture media were measured by gas chromatography after methanization, and CO_2 concentrations were calculated using the Henderson-Hasselbalch equation.

Pull-down assay

Cells were grown photoautotrophically in MOPS-P medium under the appropriate CO_2 conditions (HC or VLC) until OD_{730} = 0.4–0.7. Typically, 1–3 L of culture was harvested at 4°C by centrifugation (3,000 g, 5 min). Pellets were resuspended in suspension buffer (300 mM Tris-HCl [pH 7.5], 300 mM KCl, 15 mM $MgCl_2$) supplemented with 3×protease inhibitor cocktail (EDTA-free, Roche). Cells were disrupted by sonication (Handy Sonicator UR-20P, TOMY; amplitude setting 8, 8 cycles of 5 s ON/3 s OFF on ice).

Lysates were clarified by sequential centrifugation at 10,000 g for 10 min and 100,000 g for 20 min at 4°C, followed by filtration through a 0.22 μm membrane. The protein concentration was determined by Bradford assay and adjusted to 5 mg mL^{-1}. For the pull-down, M2 FLAG Affinity Gel (Sigma-Aldrich) was pre-equilibrated in base buffer (100 mM

Tris-HCl [pH 7.5], 5 mM $MgCl_2$, 0.1% Triton X-100). The lysate (typically 10 mg total protein) was incubated with the gel (4 μL slurry per 10 mg protein) at 4°C for 2 h with gentle rotation. After binding, the gel was washed five times each with buffers of increasing ionic strength (100, 500, and 1,500 mM KCl in base buffer). Bound proteins were eluted with 500 ng μL^{-1} of 3×FLAG peptide (Sigma-Aldrich) in base buffer by overnight rotation at 4°C. Eluates were collected by centrifugation (500 g, 1 min, 4°C) and pooled for downstream analyses (SDS-PAGE and mass spectrometry).

Co-immunoprecipitation with anti-CBP1 antibody

Cells were harvested at mid-log phase (OD_{730}=0.3–0.6) and resuspended in PBS containing 0.1% (v/v) Triton X-100, Complete Protease Inhibitor Cocktail (Roche), and Phosphatase Inhibitor Cocktail (EDTA-free, Nacalai Tesque). Cells were disrupted by sonication (UR-20P, TOMY) on ice for 1 min (6 cycles of 7 s ON/ 3 s OFF). The lysate was clarified by centrifugation at 10,000 g for 10 min and then at 100,000 g for 20 min at 4°C, followed by filtration through a 0.22 μm membrane (Roche). The protein concentration was adjusted to 5 mg mL^{-1}. For immunoprecipitation, 50 μL of Dynabeads Protein A (Invitrogen) were incubated with 10 μL of anti-CBP1 antibody in 200 μL of PBS-T for 10 min at room temperature. After washing with PBS-T, the beads were incubated with 1 mL of the cell lysate for 10 min at room temperature. The beads were washed several times with PBS, and bound proteins were eluted with 20 μL of 50 mM glycine (pH 2.8). The eluates were analyzed by immunoblotting using anti-CBP1 and anti-CCM1 antibodies.

Generation of anti-CBP1 antibody

To generate a polyclonal antibody against CBP1, the cDNA sequence encoding full-length *CBP1* was cloned into the pGEX-6P-1 vector (GE Healthcare) to produce an N-terminal GST-tagged fusion protein. The construct was transformed into *E. coli* BL21 cells. Gene expression was induced by adding IPTG to a final concentration of 1 mM when the optical density at 600 nm reached 0.8, followed by incubation for 12 h. The recombinant protein was purified using Glutathione Sepharose 4B, and the GST tag was cleaved using PreScission Protease (GE Healthcare). The resulting CBP1 protein was resolved by SDS-PAGE, and the specific band was excised from the gel and used as the antigen. Polyclonal antibodies were raised in rabbits, and the antiserum was affinity-purified using the recombinant CBP1 protein.

Construction of BiFC vectors

To visualize protein-protein interactions by bimolecular fluorescence complementation (BiFC), cDNA fragments corresponding to CCM1 and CBP1 were cloned into Gateway entry vectors (Thermo Fisher Scientific) using In-Fusion HD Cloning (Takara). Each gene was fused in-frame with either the N-terminal fragment (nEYFP, amino acids 1–174) or C-terminal fragment (cEYFP, amino acids 175–238) of enhanced yellow fluorescent protein (EYFP) split at the Asp-174/Gly-175 position. LR recombination was subsequently performed to generate destination vectors for transient expression. As negative controls, Arabidopsis thaliana nuclear proteins GLK1 and BPG4 were used (2).

Agrobacterium-mediated *Nicotiana benthamiana* leaf infiltration

Agrobacterium tumefaciens strain carrying the BiFC constructs was grown in YEP medium containing the appropriate antibiotics. Bacterial cells were harvested by centrifugation, resuspended in infiltration buffer to OD_{600} = 0.8, and incubated at room temperature for 1–2 h. The suspensions were syringe-infiltrated into the abaxial side of *N. benthamiana* leaves according to Miyaji et al. 2025 (3). Two to three days post-infiltration, small leaf sections were excised and observed under a confocal laser scanning microscope (see below).

Subcellular localization analysis of CCM1 and CBP1

To observe the subcellular localization of CCM1 and CBP1 in *Chlamydomonas*, genomic regions of CCM1 and CBP1, including their ~2.0 kb upstream sequences, were first cloned into pENTR™ 1A Dual Selection Vector (Thermo Fisher Scientific) using In-Fusion® HD Cloning Kit (Takara Bio). These entry clones were then recombined with destination vectors containing mGold through Gateway LR reactions (Thermo Fisher Scientific) to generate expression constructs. The resulting constructs were transformed into the relevant mutant backgrounds (e.g., *ccm1*-1 or *cbp1*-1) by electroporation (4).

Transgenic cells were grown under either HC or VLC conditions, then harvested at mid-log phase. For fluorescence microscopy, cells were placed on 1.0% agarose pads and sealed under coverslips. Confocal images were acquired using a Leica SP8 confocal microscope with a 63×oil-immersion objective. mGold fluorescence was excited at 543 nm, and emission was detected between 560 and 600 nm. Images were processed in LAS X software (Leica) for minor adjustments of brightness and contrast.

Measurement of photosynthetic O_2-evolving activity

Cells were harvested by centrifugation (1,000 g, 5 min, 25°C) and resuspended in Ci-depleted 20 mM HEPES–NaOH (pH 7.8) at a final chlorophyll concentration of 10–20 µg mL^{-1}. Oxygen evolution rates were measured at 25°C using a Clark-type oxygen electrode (Hansatech Instruments) under saturating white light (~700 µmol photons m^{-2} s^{-1}) as described previously (5). $NaHCO_3$ was injected stepwise to vary the Ci concentration. $K_{0.5}$(Ci) values, defined as the Ci concentration required for half-maximal O_2 evolution (V_{max}), was calculated by fitting the data to the Michaelis–Menten equation.

For acetazolamide (AZA) treatments, AZA was dissolved in DMSO at 5 mM and added to the cell suspension at a final concentration of 1% (v/v). Control samples received 1% DMSO only.

CRISPR-Cas9 system-mediated generation of mutants

To generate *cbp1*-1, we designed a guide RNA (5′-TGAGTTCTGAACGTTTGTCG-3′) targeting the second exon of the *CBP1* gene using CRISPOR (http://crispor.tefor.net/) (6). The crRNA and tracrRNA were chemically synthesized (Integrated DNA Technologies) and mixed with recombinant Cas9 protein to form a ribonucleoprotein (RNP) complex. RNP and the *AphVII* cassette (conferring hygromycin resistance) were co-introduced into WT C9 cells by electroporation (7).

Transformants were selected on TAP plates containing 30 µg mL^{-1} hygromycin and screened by colony PCR. Specific genomic insertion of the *AphVII* cassette was verified by PCR with primers 5′-GTTGTAGGTGGGTTGGAGGG-3′ (forward) and 5′-ATGTCCTTCGCCTTCTCAGC-3′ (reverse). Immunoblotting with a CBP1-specific antibody confirmed the loss of CBP1 protein in the resulting mutant.

Immunoblot analysis

Harvested cells (OD_{730} = ~0.6) were pelleted by centrifugation at 2,000 g for 5 min, then resuspended in SDS loading buffer (50 mM Tris-HCl [pH 8.0], 25% glycerol, 2% SDS, 100 mM DTT). After incubation at 37°C for 30 min, lysates were clarified by centrifugation (13,000 g, 10 min). Approximately 10 µg of total protein per lane was separated on SDS-PAGE and transferred onto a PVDF membrane (Fluoro Trans, Pall Life Science) using a semidry blotter.

Membranes were blocked in 5% (w/v) skim milk (Wako) in PBS at room temperature for 1 h, followed by washing in PBS containing 0.1% Tween-20 (PBS-T). The following

primary antibodies were used: anti-HLA3 (1:1,250) (8), anti-LCIA (1:5,000) (8), anti-LCI1 (1:5,000) (9), anti-LCIB (1:5,000) (10), anti-CBP1 (1:5,000), anti-CAH1/2 (1:2,500) (11), anti-CAH3 (1:2,000; Agrisera, AS05 073), anti-CCM1 (1:2,500) (12), and anti-Histone H3 (1:20,000; abcam, ab1791). HRP-conjugated goat anti-rabbit IgG (Invitrogen, 65-6120) was used as secondary antibody at 1:10,000 dilution.

Generation of complemented strains

For complementation of *cbp1*-1, a *CBP1* genomic fragment containing approximately 2.0 kb of the upstream promoter region was amplified using primers F2 (5′-GAAGGTGTTTGTGATACCTGCTGTG-3′) and R2 (5′-GATAATTGCATGTGTAGCTCAGTAGGAC-3′) (Fig. 2A). The amplified fragment was co-transformed with the paromomycin resistance gene (*aphVIII*) cassette into *cbp1*-1 cells by electroporation using a NEPA 21 electroporator (NEPAGENE). Transformants were selected on TAP agar plates containing 30 μg mL^{-1} paromomycin. Colonies were screened by genomic PCR using primers F1 and R1 (Fig. 2A) to identify transformants showing the WT-size 1.0 kb band. Complementation was verified by immunoblot analysis to confirm CBP1 protein accumulation, and a representative transformant was designated *cbp1*-1:*CBP1*.
For *ccm1*-1 complementation, a plasmid containing a 5.1-kb genomic fragment from pKI4XA (8), which harbors the functional CCM1 gene with its promoter region, was introduced into *ccm1*-1 by electroporation. Transformants were selected by their ability to grow photoautotrophically on MOPS-P agar plates under 0.01% CO_2, a condition non-permissive for the *ccm1*-1 mutant. A transformant showing WT-like growth and Ci affinity was selected as *ccm1*-1:*CCM1* (Table S5).

SI References

1. D. Shimamura, T. Yamano, Y. Niikawa, D. Hu, H. Fukuzawa, A pyrenoid-localized protein SAGA1 is necessary for Ca^{2+}-binding protein CAS-dependent expression of nuclear genes encoding inorganic carbon transporters in *Chlamydomonas reinhardtii*. *Photosynth. Res.* **156**, 181–192 (2023).
2. R. Tachibana et al., BPG4 regulates chloroplast development and homeostasis by suppressing GLK transcription factors and involving light and brassinosteroid signaling. *Nat. Commun*. 15, 370 (2024).
3. T. Miyaji et al., BIL7 enhances plant growth by regulating the transcription factor BIL1/BZR1 during brassinosteroid signaling. *Plant J.* **121**, e17212 (2025).

4. T. Yamano, H. Iguchi, H. Fukuzawa, Rapid transformation of *Chlamydomonas reinhardtii* without cell-wall removal. J. Biosci. Bioeng. **115**, 691–694 (2013).
5. T. Yamano, K. Miura, H. Fukuzawa, Expression analysis of genes associated with the induction of the carbon-concentrating mechanism in *Chlamydomonas reinhardtii*. *Plant Physiol.* **147**, 340–354 (2008).
6. J.P. Concordet, M. Haeussler, CRISPOR: intuitive guide selection for CRISPR/Cas9 genome editing experiments and screens. *Nucleic Acids Res.* **46**, W242–W245 (2018).
7. Y. Tsuji et al., YAK1-type protein kinase, triacylglycerol accumulation regulator 1, in the green alga *Chlamydomonas reinhardtii* is a potential regulator of cell division and differentiation into gametes during photoautotrophic nitrogen deficiency. *J Gen Appl Microbiol.* **69**, 1–10 (2023).
8. T. Yamano et al., Characterization of cooperative bicarbonate uptake into chloroplast stroma in the green alga *Chlamydomonas reinhardtii*. *Proc Natl Acad Sci U S A.***112**, 7315–7320 (2015).
9. N. Ohnishi N, et al., Expression of a low CO_2-inducible protein, LCI1, increases inorganic carbon uptake in the green alga *Chlamydomonas reinhardtii*. *Plant Cell* **22**, 3105–3117 (2010).
10. T. Yamano et al., Light and low-CO_2-dependent LCIB-LCIC complex localization in the chloroplast supports the carbon-concentrating mechanism in *Chlamydomonas reinhardtii*. *Plant Cell Physiol.* **51**, 1453–1468 (2010).
11. A. Tachiki, H. Fukuzawa, S. Miyachi, Characterization of carbonic anhydrase isozyme CA2, which is the CAH2 gene product, in *Chlamydomonas reinhardtii*. *Biosci Biotechnol Biochem.* **56**, 794–798 (1992).
12. T. Kohinata, H. Nishino, H. Fukuzawa, Significance of zinc in a regulatory protein, CCM1, which regulates the carbon-concentrating mechanism in *Chlamydomonas reinhardtii*. *Plant Cell Physiol.* **49**, 273–283 (2008).

Fig.S1

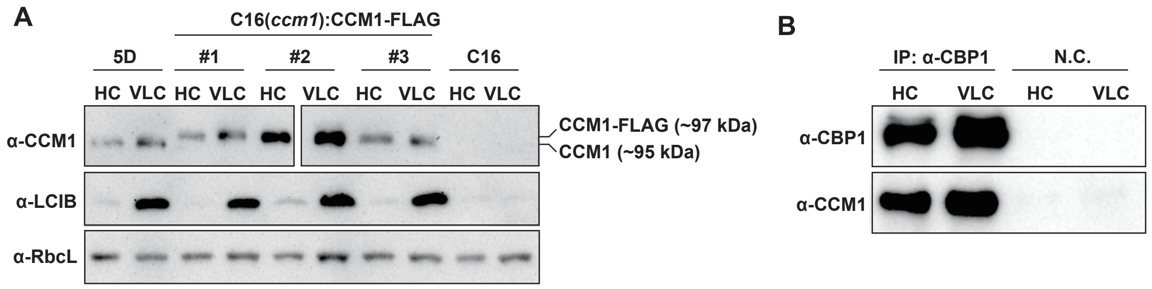

Fig.S1. Generation of CCM1-FLAG lines for MS analysis and verification of the CCM1–CBP1 interaction.
(A) To select a functionally complemented line for subsequent analysis, the CCM1-FLAG construct was introduced into the ccm1 mutant strain (C16). Three independent transformants, designated C16(*ccm1*):CCM1-FLAG #1, #2 and #3, were analyzed by immunoblotting. Functional complementation was confirmed by the restored accumulation of the CCM component LCIB under very-low-CO_2 (VLC) conditions. Among these lines, CF-2 showed the highest accumulation of both CCM1-FLAG and LCIB and was therefore selected for the pull-down assay. Cells pre-grown at 5 % CO_2 were shifted for 12 h either to the same high-CO_2 condition (HC, 5 %) or to very-low-CO_2 air (VLC, 0.04 %). RbcL served as the loading control. **(B)** Co-immunoprecipitation of CCM1 with CBP1. Total protein extracts from cells grown as in (A) were subjected to immunoprecipitation (IP) with anti-CBP1 antibodies; precipitates were analysed by immunoblotting with anti-CCM1 and anti-CBP1. “N.C.” denotes the negative control in which no antibody was added to the IP reaction.

Fig.S2

```
    >CCM1-FLAG (721 aa)
  1 MEALDAQDSL QLDVVSPSAR PAAAGGDKRD PERFYCPYPG CNRSFAELWR LKVHYRAPPD
 61 IRGSGKERGH GTELTHCPKC GKTLKPGKHH VGCSGGKSAP RQTASKRNRT GADDADEAVP
121 GSPHSKHVRG TDMDGDPHKS WQDFALTHAG YAIGAPAMLA PLKQEHPEWP PTVPQGVFVG
181 HGDRVSWLPG QVNGFVPQLQ PQRYQQPQFP PELAQAFAAA GTHAPHVYAQ QVPFASIPGY
241 PGQPGVATLQ VTTESGQVLS IPANMAGMPP GMAGLPGTLV YHQQPPPHDA AASYLAQAQA
301 HAQHAAAMHA VNSAHAQQQQ QQQQQQQQQQ PGVPAAPPAV PGVHDGMPPG TVAAAAAAAA
361 AAAAVGGSAP SALQTDVGGR PGAALPPQAA PGTGAGQGAG APAGAADGGA APAAGDAAAS
421 GGAKPVADED NLGTVFDDVE EFTRDFGRIP SPPPLPPDFH TAATGGNGML FNFSQFGQKL
481 PRTQSHTRLD RSLSAVGLGH LDVGVDGDVM YDHTDDGDLM QLLFGVPDEL PTMATIHLHK
541 WSNEEDEDDD AAEPGGGGAA AAGGGGGAAA GAGGEGGGGA GAGGGGAGAG AGEANAAAGR
601 GGAGPGPGLE AGGGGGGGGA GEGGPGAGQQ PPHHQQSVGG HDQRPLNGKT LHGHDASLAV
661 LPAPGGKSLM NGGAGHAGEE HHRDHLLDAE TFRLLQSCDD YKDHDGDYKD HDIDYKDDDD
    K

    >CBP1 (606 aa)
  1 MASAGATAPT NVQNSQKTPV TIITGFLGAG KTTLLNYILK EKGSRSIAVI ENEFGEVNID
 61 RELVAANLLA KEDLVSLENG CVCCSLRKDI VKAFAEIERR SRQAGGKKVD AIVLETTGLA
121 DPAPVAFTFF ANPWIASRFR LDSIICVVDA RYLMQHLEDG KHSDGTVNEA VQQIAFADLI
181 LLNKIDLVNS EEQKKQVLGA IRAVNNSARI VECQLNQETG RPHMDMLLFN NLFSVNRVLE
241 QIDPQFLDSD SDDDAEDEEE APSPQSGTQA QQDQASRAAK GQAAEPGAAA GADAGAGPGS
301 KATAEASAGS KAGAGCCGSG EGKGKAAVED EAAARTAGEA GPSGSSAADA KAKLLQKYAD
361 KGSVAGHKHT RDDNCEDNCE ECHIVDGMPI KGERNPKRRA KRLHDLSDVS SVGIMARGPL
421 DEYRFNMYMR DLLAEKAKDI FRCKGVLSVH GYGSTKFVFQ GVHETICYGP AEQPWKPEEQ
481 RVNQVVFIGR GLNRKALIEG FRTCVWVPLP DGWDEFRDTT TKQPFYVNRN TGEKSWTRPE
541 IACARVVATQ GKTQQPSQLL PRRTASTVGQ LALQQAAAAA ASGAVAPATA SAKAGGAASA
601 ATEVAS

    >GDH1 (448 aa)
  1 MEARTVSVLA GLAHRVASAA WRSASGLRTC TTTTPTYGRT SVYVKEALDL LDFDPQVEKA
 61 ILNPDREVTV NLVVPMDNGE VNMFPAYRVQ HNNALGPFKG GIIYHPGVTL ENMRNLASLN
121 TWKFSLLNVQ FGGAKGGVGV DPRSLSERET EKLTRKYVQA LQEVIGPHTD IPAPDINTDE
181 HHMAWIFDQY SRLRGFAPAA VTGKPTWLHG IVGRDKAGGR GAAIATREFL TRSLRRKVAG
241 TSFLIQGFGK LGSWTAQILQ QEMGAKIVGV SCSETAVYNE EGLDIPALRA HVAAGGLLKD
301 FPGGTGVLND DSFLDLPADV FIPCAVDGTI HAGNVHRCVN FKAVVEAANG ALTPEADAAL
361 RKAGVPVLPD LIANGGAVVV SFFEWVQNNQ NMQWEEDDVK RELDRYLTDA FEALLREQSL
421 HAGCSLRTAG YLVALRRLQQ ADSVRGHS

    >GDH2 (449 aa)
  1 MASALLACGR QLSCALGGLS LGHQALGAIG GAFRRHASSH AENTNTFLRE ALVKLDYPEK
 61 LQNLLLTPRR EMSVELVVQM DDGQIEVFNA YRVQHNNARG PYKGGLRYHP QVDLDDVRSL
121 ASLMTWKTAV MDIPYGGAKG GVTVDPRKLS ERELEKMTRK LVVAIKEIIG TYEDIPAPDM
181 NTDAKVMAWF FDEYSKYKGF SPGVVTGKPV YLHGSLGREA ATGRGTTFAI RELLKALHMG
241 KIADQKYVIQ GFGNVGAWAA QLLWEAGGKV VAISDVAGAV HNEQVRGLDI GALRKHVASG
301 KPLAEFTGGA AVPKQDILLH PCDVLIPAAI GGVIGPEEAK KLQCKVVVEA ANGPTTPEGD
361 MVLRDRGITV LPDIYTNGGG VTVSFFEWVQ NLQNFKWEED DVNRKLDRKM ADAFAALWAV
421 HKEMNVPLRT AAFVVALQRV TRAEVHRGFD
```

Fig.S2. Sequence coverage of CCM1, CBP1, GDH1 and GDH2 obtained by LC-MS/MS.
Full-length amino-acid sequences are shown for each protein, with regions identified by mass spectrometry highlighted in red. Sequence coverage amounts to 37.1 % for CCM1, 53.0 % for CBP1, 31.5 % for GDH1 and 52.3 % for GDH2. In the CCM1 panel, three serine phosphorylation sites are annotated: S122 and S451 (boxed in black) were detected under both high-CO_2 and very low-CO_2 conditions, whereas S10 (boxed in red) was observed only under very low-CO_2 (VLC) conditions. Residues derived from the C-terminal FLAG tag are shown in blue.

Fig.S3

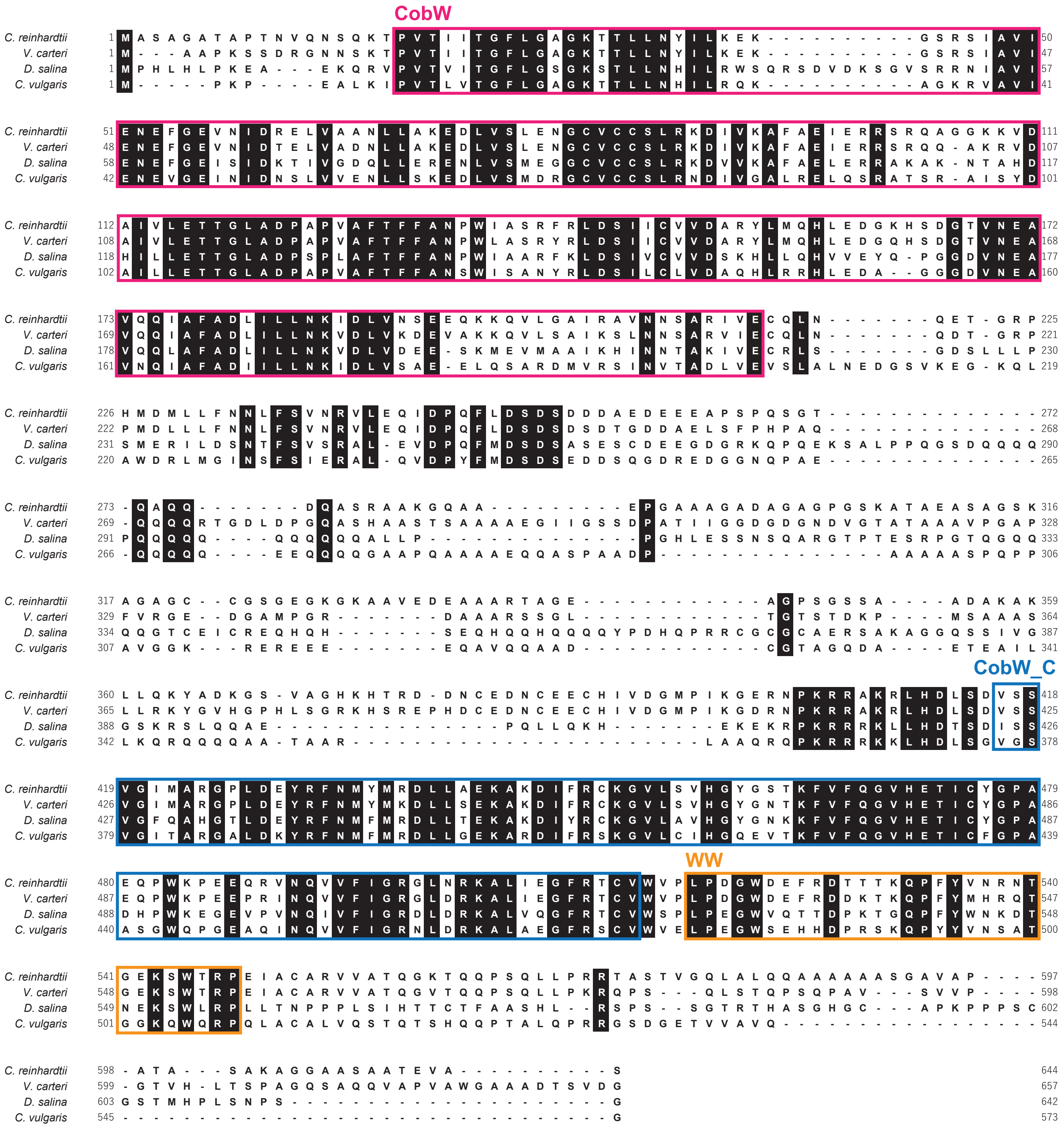

Fig. S3. Multiple sequence alignment of CBP1 proteins from various green algae species. The alignment includes sequences from *Chlamydomonas reinhardtii*, *Volvox carteri*, *Dunaliella salina*, and *Chlorella vulgaris*. Identical amino acids are highlighted in black. The alignment reveals conserved regions across the different species, particularly in the CobW ,CobW_C and WW domains. Numbers on the right indicate the amino acid positions. Gaps in the alignment are represented by dashes. The alignment was performed using MEGAX.

Fig.S4

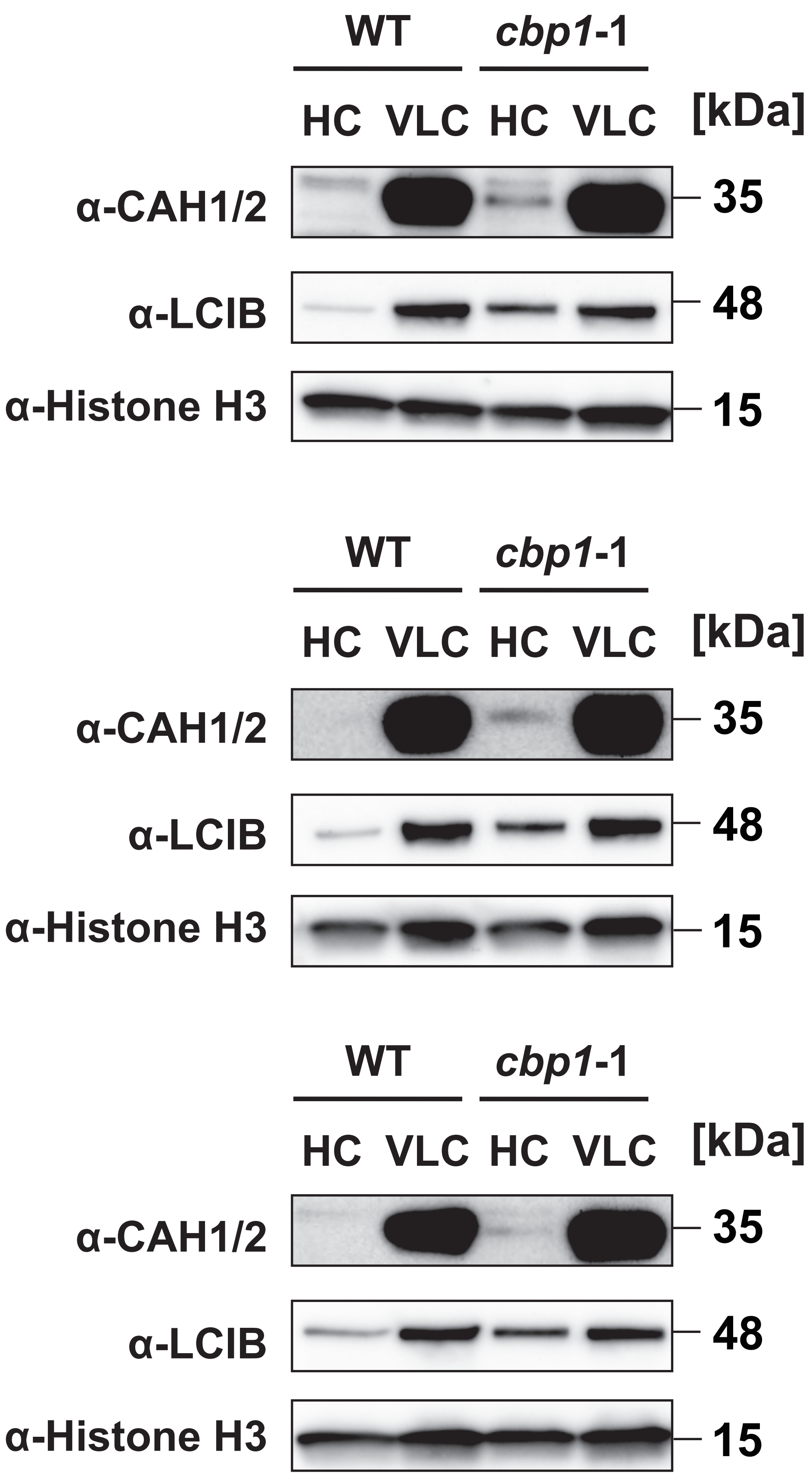

Fig.S4. Accumulation of CAH1 and LCIB in WT and *cbp1*-1.
Cells were first grown under 5% (v/v) CO_2 condition for 24 h and shifted to 5% (v/v) CO_2 (HC) or 0.04% (v/v) CO_2 (VLC) conditions for 12 h. Western blot analysis was performed using antibodies against the indicated proteins from three bioligical replicates. Histone H3 was used as a loading control.

Table S1. Proteins identified by LC-MS/MS from immunoprecipitated gel bands.

Sample name		Reference	P (pro)	Sf	Score	Coverage	MW	Peptide (Hits)
01_CCM1	1	estExt_fgenesh2_pg.C_50429 {CCM1} regulator of CO2-responsive genes	7.07E-13	7.50	80.30	21.30	70033.89	9 (9 0 0 0 0)
	2	estExt_gwp_1H.C_900015 {ADH1}	1.02E-08	0.94	10.17		102168.10	1 (1 0 0 0 0)
02_74k	1	RWI_chlre3.14.27.2.51 Nickel chaperone for hydrogenase or urease, putative	3.28E-13	14.43	150.32	34.10	64549.51	15 (15 0 0 0 0)
	2	estExt_fgenesh2_pg.C_50429 {CCM1} regulator of CO2-responsive genes	6.05E-12	0.98	10.29		70033.89	2 (2 0 0 0 0)
	3	Chlre2_kg.scaffold_1000769	5.86E-07	0.96	10.17		75461.40	1 (1 0 0 0 0)
	4	Chlre2_kg.scaffold_1000770	1.66E-07	0.95	10.16		67702.49	1 (1 0 0 0 0)
03_45k	1	estExt_GenewiseW_1.C_760022 {GDH2} glutamate dehydrogenase	4.76E-10	3.75	40.23	9.30	49004.55	4 (4 0 0 0 0)
	2	estExt_fgenesh1_pm.C_440001 {IDA5} Actin	6.30E-06	1.89	20.19		41808.93	2 (2 0 0 0 0)
	3	fgenesh2_pg.C_scaffold_2000011	2.58E-05	0.93	10.18		44510.41	1 (1 0 0 0 0)

Proteins present in three gel slices—whole CCM1 immunoprecipitate (01_CCM1), the ~75 kDa band (02_74k), and the ~45 kDa band (03_45k)—were excised, digested with trypsin, and analysed by LC-MS/MS. Mass spectra were acquired on a Q-TOF instrument (Waters) at NAIST and on an LTQ ion-trap (Thermo Scientific) at Okayama University, and searched with Mascot or BioWorks against the Chlamydomonas reinhardtii protein database (JGI v3.1). Columns are as follows. Reference: JGI protein ID and annotation returned as the top hit. P (pro): ranking of the hit within the sample. Sf: Mascot/BioWorks scoring function (lower E-value or higher score indicates greater confidence). Score: confidence score assigned by the search programme. Coverage (%): percentage of the predicted protein sequence covered by matched peptides. MW (kDa): theoretical molecular mass calculated from the predicted amino-acid sequence. Peptide (Hits): number of unique tryptic peptides (and total spectra) matched to the protein.

Table S2. Table S2 Tryptic peptides identified by LC-MS/MS from the CCM1 immunoprecipitate and the 75 kDa (P75) and 45 kDa (P45) gel slices.

						P (pro)	Sf	Score	Coverage	MW		Peptide (Hits)	Comments
Sample name		Peptide	MH+	DeltaM	z	P (pep)	Sf	XC	DeltaCn	Sp	RSp	Ions	
01_CCM1	1	estExt_fgenesh2_pg.C_50429 {CCM1} regulator of CO2-responsive genes				7.07E-13	7.50	80.30	21.30	70033.89		9 (9 0 0 0 0)	
		K.TLHGHDASLAVLPAPGGK.S	1740.94	1.00	2	1.10E-08	0.97	4.21	0.54	1454.97	1.0	22/34	
		R.PGAALPPQAAPGTGAGQGAGAPAGAADGGAAPAAGDAAASGGAK.P	3479.69	1.76	3	7.07E-13	0.95	4.78	0.55	1408.47	1.0	47/172	
		R.TGADDADEAVPGS*PHSK.H	1733.70	1.06	2	1.97E-06	0.88	3.89	0.18	762.46	1.0	28/48	phosphorylated fragment
		R.TGADDADEAVPGSPHSK.H	1653.74	1.96	2	5.71E-08	0.95	4.03	0.50	753.75	1.0	22/32	
		R.VSWLPGQVNGFVPQLQPQR.Y	2150.15	1.58	3	3.52E-08	0.89	2.97	0.30	1434.91	1.0	31/72	
		R.DHLLDAETFR.L	1216.60	0.18	2	5.84E-07	0.93	2.81	0.37	1210.01	1.0	15/18	
		K.QEHPEWPPTVPQGVFVGHGDR.V	2369.14	1.73	3	5.86E-07	0.95	4.80	0.46	1103.86	1.0	32/80	
		K.PVADEDNLGTVFDDVEEFTR.D	2268.03	0.64	2	3.21E-12	0.98	5.91	0.56	2470.27	1.0	28/38	
		K.PVADEDNLGTVFDDVEEFTR.D	2268.03	1.90	3	3.20E-07	0.96	4.85	0.48	1519.17	1.0	29/76	
	2	estExt_gwp_1H.C_900015 {ADH1}				1.02E-08	0.94	10.17		102168.10		1 (1 0 0 0 0)	
		R.APATDEALTELK.A	1258.65	0.99	2	1.02E-08	0.94	3.50	0.44	931.93	1.0	17/22	
02_74k	1	RWI_chlre3.14.27.2.51 Nickel chaperone for hydrogenase or urease, putative				3.28E-13	14.43	150.32	34.10	64549.51		15 (15 0 0 0 0)	
		R.TAGEAGPSGSSAADAK.A	1376.63	0.46	2	3.69E-11	0.96	3.75	0.56	1319.44	1.0	23/30	
		K.AAVEDEAAAR.T	1002.49	1.12	2	1.82E-05	0.96	3.47	0.44	1426.02	1.0	16/18	
		K.GQAAEPGAAAGADAGAGPGSK.A	1710.80	1.30	2	3.28E-13	0.98	6.19	0.58	1728.52	1.0	32/40	
		K.IDLVNSEEQK.K	1174.59	0.98	2	1.78E-06	0.95	3.56	0.44	1050.29	1.0	16/18	
		K.GVLSVHGYGSTK.F	1204.63	0.76	2	1.87E-07	0.95	3.17	0.58	1132.18	1.0	18/22	
		K.IDLVNSEEQKK.Q	1302.69	1.13	2	9.53E-07	0.95	3.71	0.41	912.46	1.0	17/20	
		R.YLMQHLEDGK.H	1233.59	0.60	2	5.22E-07	0.96	3.23	0.56	1289.49	1.0	16/18	
		R.SIAVIENEFGEVNIDR.E	1804.91	1.01	2	2.76E-11	0.98	5.34	0.51	2927.35	1.0	25/30	
		K.TQQPSQLLPR.R	1167.65	0.50	2	1.83E-05	0.93	3.04	0.42	817.40	1.0	15/18	
		R.VNQVVFIGR.G	1031.60	1.02	2	9.95E-06	0.96	3.85	0.38	1460.08	1.0	15/16	
		R.LHDLSDVSSVGIMAR.G	1599.82	1.15	2	1.64E-12	0.98	5.21	0.62	2366.77	1.0	24/28	
		R.ELVAANLLAK.E	1041.63	0.58	2	2.12E-05	0.93	2.43	0.41	1200.07	1.0	16/18	
		R.TASTVGQLALQQAAAAAASGAVAPATASAK.A	2654.41	1.24	2	6.76E-11	0.98	6.47	0.56	1802.05	1.0	29/58	
		K.HSDGTVNEAVQQIAFADLILLNK.I	2496.31	1.21	3	5.92E-06	0.96	4.59	0.52	1387.03	1.0	37/88	
		K.TPVTIITGFLGAGK.T	1374.80	1.22	2	3.00E-10	0.97	4.49	0.54	1762.81	1.0	21/26	
	2	estExt_fgenesh2_pg.C_50429 {CCM1} regulator of CO2-responsive genes				6.05E-12	0.98	10.29		70033.89		2 (2 0 0 0 0)	carry-over from the previous analysis
		K.PVADEDNLGTVFDDVEEFTR.D	2268.03	0.81	2	6.05E-12	0.98	5.89	0.56	2406.99	1.0	29/38	
		K.PVADEDNLGTVFDDVEEFTR.D	2268.03	1.06	3	2.27E-05	0.92	3.86	0.40	1276.99	1.0	27/76	
	3	Chlre2_kg.scaffold_1000769				5.86E-07	0.96	10.17		75461.40		1 (1 0 0 0 0)	
		R.SAVEGAEAGEGNGAGR.S	1431.65	1.46	2	5.86E-07	0.96	3.40	0.42	1935.81	1.0	23/30	
	4	Chlre2_kg.scaffold_1000770				1.66E-07	0.95	10.16		67702.49		1 (1 0 0 0 0)	
		K.LVGFDTSEPTAR.N	1292.65	1.10	2	1.66E-07	0.95	3.22	0.49	1182.64	1.0	17/22	
03_45k	1	estExt_GenewiseW_1.C_760022 {GDH2} glutamate dehydrogenase				4.76E-10	3.75	40.23	9.30	49004.55		4 (4 0 0 0 0)	
		K.WEEDDVNR.K	1062.45	1.32	2	1.68E-05	0.92	2.72	0.43	705.23	1.0	13/14	
		R.YHPQVDLDDVR.S	1356.65	1.21	2	5.62E-07	0.89	2.85	0.32	612.33	1.0	16/20	
		K.PVYLHGSLGR.E	1098.61	0.97	2	1.27E-07	0.96	3.09	0.50	1316.45	1.0	17/18	
		K.PLAEFTGGAAVPK.Q	1257.68	1.23	2	4.76E-10	0.98	4.60	0.57	1695.21	1.0	21/24	
	2	estExt_fgenesh1_pm.C_440001 {IDA5} Actin				6.30E-06	1.89	20.19		41808.93		2 (2 0 0 0 0)	
		K.DSYVGDEAQSK.R	1198.52	0.92	2	6.30E-06	0.94	2.91	0.46	1053.98	1.0	17/20	
		R.GYSFTTTAER.E	1132.53	0.86	2	1.48E-05	0.95	3.04	0.53	907.78	1.0	16/18	
	3	fgenesh2_pg.C_scaffold_2000011				2.58E-05	0.93	10.18		44510.41		1 (1 0 0 0 0)	
		K.EYVVQEGDVLLFR.F	1566.82	1.47	2	2.58E-05	0.93	3.58	0.43	732.49	1.0	17/24	

Each band observed in Fig.1 was excised, digested with trypsin, and analysed by LC-MS/MS on a Q-TOF (Waters) or LTQ ion-trap (Thermo Scientific). For every sample the table lists all peptide-spectrum matches that passed the significance thresholds of Mascot (NAIST) or BioWorks (Okayama Univ.). Peptides in which phosphorylation was detected are highlighted in blue.

Columns. Peptide: identified peptide sequence (asterisk denotes a post-translational modification, here phosphorylation). MH^+: monoisotopic mass of the protonated peptide (Da). ΔM: mass error relative to the theoretical value (Da). z: precursor ion charge state. P (pro)/P (pep) and Sf: Mascot or BioWorks probability and score for the corresponding protein (P (pro)) or individual peptide (P (pep)). XC: cross-correlation score. ΔCn: normalised difference between the best and second-best matches. Sp and RSp: preliminary score and rank of preliminary score. Coverage and MW for each protein are reproduced from Table S1. Ions: number of matched fragment ions (y + b) over total considered.

Table S3. Proteins co-purifying with CCM1-FLAG under high- and very-low-CO_2 conditions, identified by in-solution tryptic digestion.

Reference	P (pro)	Sf	Score	Coverage	MW	Peptide (Hits)
High-CO2 conditions						
jgi|Chlre3|195946|RWI_chlre3.14.27.2.51 Nickel chaperone for hydrogenase or urease, putative	3.33E-15	18.96	200.35	45.90	64549.51	27 (27 0 0 0 0)
jgi|Chlre3|82916|estExt_GenewiseW_1.C_760022 {GDH2} glutamate dehydrogenase	1.72E-13	8.46	90.27	24.90	49004.55	9 (9 0 0 0 0)
jgi|Chlre3|186972|estExt_fgenesh2_pg.C_50429 {CCM1} regulator of CO2-responsive genes	6.27E-13	7.58	80.29	25.50	70033.89	8 (8 0 0 0 0)
jgi|Chlre3|190969|estExt_fgenesh2_pg.C_220037 {GDH1}	1.74E-08	5.69	60.25	19.00	48534.99	6 (6 0 0 0 0)
jgi|Chlre3|134382|estExt_gwp_1W.C_30492 {EF1A1} eukaryotic translation elongation factor 1 alpha 1	1.17E-09	3.64	40.19	11.40	50792.30	4 (4 0 0 0 0)
jgi|Chlre3|137452|estExt_gwp_1W.C_220126 {HSP70C} Heat shock protein 70C	1.22E-12	2.91	30.25	8.70	65263.79	3 (3 0 0 0 0)
jgi|Chlre3|188942|estExt_fgenesh2_pg.C_120244 {IF4A} Similar to Eukaryotic Initiation Factor 4A	5.03E-12	1.94	20.21	7.00	47024.02	2 (2 0 0 0 0)
jgi|Chlre3|128745|estExt_gwp_1H.C_150086 {RCA} rubisco activase	2.04E-13	1.87	20.25	10.80	45000.80	2 (2 0 0 0 0)
jgi|Chlre3|10441|fgenesh1_pg.C_scaffold_11000230 {FBP} fructose-1,6-bisphosphatase	6.56E-07	1.87	20.19	8.00	35372.45	2 (2 0 0 0 0)
Reference	P (pro)	Sf	Score	Coverage	MW	Peptide (Hits)
Very low-CO2 conditions						
jgi|Chlre3|195946|RWI_chlre3.14.27.2.51 Nickel chaperone for hydrogenase or urease, putative	1.67E-14	18.87	200.35	43.20	64549.5	23 (23 0 0 0 0)
jgi|Chlre3|82916|estExt_GenewiseW_1.C_760022 {GDH2} glutamate dehydrogenase	1.43E-11	11.33	120.29	34.90	49004.6	12 (12 0 0 0 0)
jgi|Chlre3|186972|estExt_fgenesh2_pg.C_50429 {CCM1} regulator of CO2-responsive genes	1.11E-16	10.51	110.34	22.90	70033.9	12 (12 0 0 0 0)
jgi|Chlre3|190969|estExt_fgenesh2_pg.C_220037 {GDH1}	1.79E-12	7.69	80.31	28.80	48535.0	8 (8 0 0 0 0)
jgi|Chlre3|191668|estExt_fgenesh2_pg.C_260094 {ICL1} isocitrate lyase	9.48E-12	3.79	40.25	19.70	45719.7	4 (4 0 0 0 0)
jgi|Chlre3|137452|estExt_gwp_1W.C_220126 {HSP70C} Heat shock protein 70C	1.38E-11	2.94	30.29	8.90	65263.8	3 (3 0 0 0 0)
jgi|Chlre3|104082|e_gwH.26.231.1	3.05E-09	3.89	40.28	65.50	8961.9	4 (4 0 0 0 0)
jgi|Chlre3|183343|estExt_fgenesh2_kg.C_140079	1.07E-11	2.83	30.24	15.30	25643.5	3 (3 0 0 0 0)
jgi|Chlre3|144137|Chlre2_kg.scaffold_10000017	4.93E-06	1.73	20.16	14.90	15907.2	2 (2 0 0 0 0)

FLAG-tagged CCM1 complexes were affinity-purified from cells cultured continuously at 5 % CO_2 (high CO_2, HC) or from cells shifted for 12 h to 0.04 % CO_2 (very-low CO_2, VLC). Eluates were reduced, alkylated, and digested with trypsin in solution before LC-MS/MS analysis on an LTQ ion-trap (Thermo Scientific) at Okayama University. Spectra were searched with BioWorks against the Chlamydomonas reinhardtii database (JGI v3.1). Only proteins represented by ≥ 2 unique peptides in at least two technical replicates for either condition are reported. Columns are as follows. Reference: JGI protein ID and annotation returned as the top hit. P (pro): ranking of the hit within the sample. Sf: Mascot/BioWorks scoring function (lower E-value or higher score indicates greater confidence). Score: confidence score assigned by the search programme. Coverage (%): percentage of the predicted protein sequence covered by matched peptides. MW (kDa): theoretical molecular mass calculated from the predicted amino-acid sequence. Peptide (Hits): number of unique tryptic peptides (and total spectra) matched to the protein. Proteins that were already identified by the earlier in-gel digestion workflow (Table S1 and S2) are highlighted in yellow to facilitate comparison between the two methodologies.

Table S4. Peptide map of the excised CCM1-FLAG band and its phosphorylation sites.

						P (pro)	Sf	Score	Coverage	MW		Peptide (Hits)	Comments
Sample name		Peptide	MH+	DeltaM	z	P (pep)	Sf	XC	DeltaCn	Sp	RSp	Ions	
CCM1-HC	1	estExt_fgenesh2_pg.C_50429 {CCM1} regulator of CO2-responsive genes											
		-.M]EALDAQDSLQLDVVSPSAR.P	2187.06	0.46	2	8.87E-11	0.95	3.57	0.54	977.3	1	24/38	
		K.PVADEDNLGTVFDDVEEFTR.D	2268.03	0.86	2	6.67E-11	0.98	6.19	0.56	1972.4	1	26/38	
		K.SWQDFALTHAGYAIGAPAMLAPLK.Q	2529.30	1.02	3	1.66E-04	0.88	3.87	0.29	982.2	1	31/92	
		K.TLHGHDASLAVLPAPGGK.S	1740.94	1.17	2	8.31E-08	0.95	3.78	0.49	1213.4	1	20/34	
		R.DHLLDAETFR.L	1216.60	0.58	2	2.03E-05	0.93	2.98	0.34	1228.6	1	15/18	
		R.IPS*PPPLPPDFHTAATGGNGMLFNFSQFGQK.L	3350.57	0.75	3	9.46E-12	0.94	5.77	0.25	999.5	1	49/180	Phosphorylated
		R.PGAALPPQAAPGTGAGQGAGAPAGAADGGAAPAAGDAAASGGAK.P	3479.69	1.39	3	2.39E-11	0.97	5.53	0.65	1499.4	1	48/172	
		R.TGADDADEAVPGS*PHSK.H	1733.70	0.98	2	5.99E-06	0.89	4.08	0.18	802.9	1	27/48	Phosphorylated
		R.VSWLPGQVNGFVPQLQPQR.Y	2150.15	1.20	2	3.15E-10	0.98	4.91	0.46	1972.9	1	27/36	
CCM1-VLC	1	estExt_fgenesh2_pg.C_50429 {CCM1} regulator of CO2-responsive genes											
		-.M]EALDAQDSLQLDVVS*PSARPAAAGGDKR.D	3090.46	1.19	3	1.71E-10	0.96	5.45	0.09	2499.9	1	57/168	Phosphorylated
		-.M]EALDAQDSLQLDVVSPSAR.P	2187.06	1.46	2	1.17E-11	0.97	4.00	0.59	1411.8	1	28/38	
		-.M]EALDAQDSLQLDVVSPSARPAAAGGDKR.D	3010.49	1.81	3	2.22E-15	0.97	5.86	0.53	1361.4	1	39/112	
		K.PVADEDNLGTVFDDVEEFTR.D	2268.03	0.99	2	3.96E-12	0.99	6.19	0.58	2364.8	1	28/38	
		K.SLMNGGAGHAGEEHHR.D	1659.74	1.28	2	7.02E-09	0.94	4.02	0.52	887.8	1	18/30	
		K.SLMNGGAGHAGEEHHRDHLLDAETFR.L	2857.32	1.34	3	1.31E-07	0.88	3.89	0.35	938.9	1	29/100	
		K.TLHGHDASLAVLPAPGGK.S	1740.94	-0.24	2	1.31E-07	0.96	3.85	0.49	1438.1	1	22/34	
		R.DHLLDAETFR.L	1216.60	0.59	2	6.90E-05	0.95	2.95	0.38	1369.6	1	16/18	
		R.IPS*PPPLPPDFHTAATGGNGMLFNFSQFGQK.L	3350.57	-0.40	3	7.05E-13	0.97	6.49	0.35	1703.0	1	52/180	Phosphorylated
		R.PGAALPPQAAPGTGAGQGAGAPAGAADGGAAPAAGDAAASGGAK.P	3479.69	0.75	3	2.11E-14	0.97	5.56	0.62	1994.2	1	53/172	
		R.SFAELWR.L	908.46	0.97	2	1.86E-04	0.88	2.10	0.42	592.4	1	11/12	
		R.TGADDADEAVPGS*PHSK.H	1733.70	0.98	2	5.99E-06	0.89	4.08	0.18	802.9	1	27/48	Phosphorylated
		R.TGADDADEAVPGSPHSK.H	1653.74	0.90	2	1.11E-07	0.93	3.83	0.48	617.0	1	21/32	
		R.VSWLPGQVNGFVPQLQPQR.Y	2150.15	0.74	2	1.19E-12	0.98	5.44	0.47	1913.3	1	27/36	

The single CCM1-FLAG band was cut from an SDS-PAGE gel after FLAG affinity purification, reduced, alkylated, and subjected to in-gel tryptic digestion. Resulting peptides were analysed by LC-MS/MS on an LTQ ion-trap (Thermo Scientific) at Okayama University, and spectra were searched with BioWorks against the Chlamydomonas reinhardtii database (JGI v3.1). The table lists every CCM1 peptide that met the BioWorks significance threshold in at least two technical replicates of either CO_2 treatment—continuous high CO_2 (5 %, HC) or a 12 h shift to very-low CO_2 (0.04 %, VLC). Peptides that contain a phosphorylation site are shaded light blue.

Table S5. Photosynthetic parameters of WT and transformant cells

Growth condition	Measuring condition	Strain name	Vmax of O_2-evolving activity [μmol O_2·mgChl^{-1}·h^{-1}]	$K_{0.5}$(Ci) [μM]
VLC	20 mM HEPES-NaOH (pH 7.8)	WT	229 ± 40	28 ± 4
VLC	20 mM HEPES-NaOH (pH 7.8)	*cbp1*-1	221 ± 26	29 ± 5
VLC	20 mM HEPES-NaOH (pH 7.8)	*cbp1*-1:*CBP1*	219 ± 15	20 ± 5
VLC	20 mM HEPES-NaOH (pH 7.8)	*ccm1*-1	224 ± 52	254 ± 111
VLC	20 mM HEPES-NaOH (pH 7.8)	*ccm1*-1:*CCM1*	217 ± 10	22 ± 4
HC	20 mM HEPES-NaOH (pH 7.8)	WT	212 ± 32	381 ± 26
HC	20 mM HEPES-NaOH (pH 7.8)	*cbp1*-1	178 ± 11	248 ± 27
HC	20 mM HEPES-NaOH (pH 7.8)	*cbp1*-1:*CBP1*	202 ± 9	475 ± 40
HC	20 mM HEPES-NaOH (pH 7.8) +1% [v/v] DMSO	WT	208 ± 11	394 ± 31
HC	20 mM HEPES-NaOH (pH 7.8) +1% [v/v] DMSO	*cbp1*-1	203 ± 23	169 ± 5
HC	20 mM HEPES-NaOH (pH 7.8) + 5 mM AZA in DMSO	*cbp1*-1	213 ± 38	416 ± 70

The data are shown ± standard error (SE), which were obtained from three independent experiments. AZA, acetazolamide; DMSO, Dimethyl sulfoxide; HC, high-CO_2; $K_{0.5}$(Ci), Ci concentration required for half of Vmax; VLC, very low-CO_2; Vmax, maximum O_2-evolving activity.

Table S6. VLC-inducible and CCM1-dependent genes differently expressed in *cbp1*-1 cells compared with those in WT and *cbp1*-1:*CBP1* cells in HC conditions

Gene ID	Gene name	Description	Average TPM in WT			Average TPM in *ccm1*-1			Average TPM in *ccm1*-1:*CCM1*			Average TPM in *cbp1*-1			Average TPM in *cbp1*-1:*CBP1*		
			HC	VLC 0.3 h	VLC 2.0 h	HC	VLC 0.3 h	VLC 2.0 h	HC	VLC 0.3 h	VLC 2.0 h	HC	VLC 0.3 h	VLC 2.0 h	HC	VLC 0.3 h	VLC 2.0 h
**Cre01.g000150**	**ZRT2**	Zinc-nutrition responsive permease transporter	3.8	32.0	8.8	1.8	0.9	0.4	4.0	24.2	11.0	13.0	30.2	8.8	4.1	27.0	8.2
**Cre01.g053950**	—	Factors identified as interacting with LCIB/LCIC in the proteome analysis by Mackinder et al. Cell (2017)	0.7	50.2	28.7	0.1	0.2	0.5	1.6	37.7	34.0	12.3	80.3	48.6	3.2	84.8	36.9
**Cre03.g151650**	***SMM7***	Putative methyltransferase that localizes to the pyrenoid matrix and is implicated in pyrenoid biogenesis (Mackinder et al. Cell 2017)	7.7	252.3	107.6	0.1	0.2	0.1	13.5	217.1	91.9	27.0	144.8	47.9	8.5	163.7	51.9
**Cre03.g162800**	***LCI1***	Low-CO2-inducible membrane protein	0.1	1270.3	947.6	0.0	0.5	0.1	0.2	991.2	1116.6	1.0	1490.1	709.4	0.1	456.1	307.1
**Cre03.g212641**	—	Putative histone acetyltransferase of the CBP family 1 (Li et al. Genomics 2020)	1.9	14.4	3.2	0.5	1.1	1.5	1.3	10.1	4.4	12.5	17.0	10.6	5.0	20.5	8.8
**Cre03.g212977**	—	Putative histone acetyltransferase of the CBP family 1 (Li et al. Genomics 2020)	1.1	40.0	31.6	0.0	0.0	0.0	1.7	25.7	45.3	18.0	54.2	38.0	5.7	43.6	28.3
**Cre04.g215900**	—	Tyrosine-protein kinase ephrin type A/B receptor-like protein	0.4	14.1	6.1	0.2	0.1	0.0	0.5	9.6	6.7	7.8	21.3	11.5	3.6	19.5	10.3
**Cre04.g215952**	—	—	0.3	2.4	2.3	0.1	0.0	0.1	0.1	1.9	1.4	11.0	37.4	21.2	4.3	28.3	16.3
**Cre04.g222750**	***CCP2***	Low-CO2-inducible membrane protein	3.8	226.0	235.4	2.0	4.1	2.7	8.1	131.3	318.9	26.2	529.9	314.0	12.5	266.7	234.0
**Cre04.g222800**	***LCID***	Low-CO2 inducible protein D	2.8	158.5	236.4	2.1	3.2	3.4	3.2	101.2	324.7	10.5	253.8	174.2	5.3	141.5	105.5
**Cre04.g223100**	***CAH1***	Periplasmic carbonic anhydrase	13.9	5174.3	8334.0	2.4	4.2	2.7	19.0	5047.9	9969.0	114.1	2696.0	8316.8	3.1	1751.1	5037.5
**Cre06.g278199**	***LOCO2***	Low CO2 sensitive 2 (Fauser et al. Nat Genet 2022)	0.9	3.1	4.0	1.4	2.3	1.2	2.0	5.9	7.9	9.0	29.5	20.9	4.5	23.5	10.3
**Cre06.g309000**	***LCIA***	Inorganic carbon channel localized at chloroplast membrane	0.6	1456.6	1270.7	0.0	0.6	0.0	0.4	990.5	1588.1	5.0	2114.9	1347.6	0.4	1189.6	813.5
**Cre07.g339000**	***RbcX-lib***	Chaperon-like RbcX protein (Bracher et al. PLOS One 2015)	1.0	18.0	11.4	0.7	0.5	0.4	2.5	14.3	12.7	8.3	29.5	16.1	3.8	23.8	12.4
**Cre08.g367400**	***LHCSR3.2***	Stress-related chlorophyll a/b binding protein 3	0.3	824.0	684.3	0.1	4.4	3.1	2.0	635.7	512.4	6.3	1569.0	686.7	1.9	957.0	399.7
**Cre08.g367500**	***LHCSR3.1***	Stress-related chlorophyll a/b binding protein 2	0.6	655.5	582.2	0.3	2.4	2.2	1.3	477.1	442.2	5.2	1354.3	616.3	1.7	782.6	337.7
**Cre09.g394473**	***LCI9***	Low-CO2 inducible protein 9 containing starch-binding domain of CBM_20 (pfam00686)	14.7	353.4	240.4	0.5	0.6	1.6	21.2	306.7	271.1	61.0	229.0	151.5	18.3	300.5	164.3
**Cre09.g395732**	—	DnaJ domain containing protein	0.8	17.6	2.1	0.6	1.9	3.1	0.7	13.9	4.3	4.4	21.7	5.2	1.5	28.4	5.5
**Cre09.g399552**	***LCR1***	Low-CO2 response regulator, Myb-like transcription factor	0.5	54.6	53.2	0.1	0.9	1.1	0.5	22.6	60.7	13.2	61.3	74.8	1.0	37.6	44.5
**Cre10.g452800**	***LCIB***	Low-CO2-inducible protein B	172.0	5242.3	901.0	58.1	181.8	187.7	202.6	5126.6	1390.4	612.7	4717.7	1372.4	149.3	4493.7	1281.3
**Cre11.g481104**	—	protein kinase family protein	1.6	22.4	8.7	1.6	2.0	1.5	1.3	16.3	12.6	6.2	24.8	10.9	3.3	25.2	8.1
**Cre16.g662600**	***BST1***	Thylakoid localized bestrophin-like protein 1	0.1	116.6	8.3	0.0	0.1	0.0	0.1	55.0	10.2	11.7	183.5	31.2	0.4	155.1	25.8
**Cre16.g663450**	***BST3/LCI11***	Thylakoid localized bestrophin-like protein 3	40.9	700.5	359.2	16.8	22.2	17.6	43.0	679.9	455.5	125.0	826.1	346.1	41.4	673.4	233.3
**Cre16.g667250**	—	—	26.9	194.5	194.6	22.7	20.9	48.9	34.6	173.9	252.4	87.8	358.2	252.8	46.4	258.3	215.3
**Cre16.g674291**	—	Protein kinase family protein	0.9	16.2	6.1	0.9	1.3	0.7	1.1	10.5	8.4	19.4	32.6	34.1	9.9	34.4	19.7
**Cre16.g685050**	***LCI15***	Cobalamin synthesis protein cobW C-terminal domain (CobW_C) and WW domain containing protein	17.9	225.5	153.1	8.9	20.0	16.9	13.6	175.7	243.3	71.5	324.9	314.5	19.3	257.4	203.3
**Cre16.g685100**	—	Cobalamin synthesis protein cobW C-terminal domain (CobW_C) and WW domain containing protein	0.5	11.6	7.9	0.0	0.0	0.1	0.3	4.4	7.7	1.8	22.1	17.9	0.4	9.2	9.9

Gene ID	WT_HC vs *cbp1*-1_HC		*cbp1*-1:*CBP1*_HC vs *cbp1*-1_HC		WT_VLC 0.3 h vs *cbp1*-1_VLC 0.3 h		*cbp1*-1:*CBP1*_VLC 0.3 h vs *cbp1*-1_VLC 0.3 h		WT_VLC 2.0 h vs *cbp1*-1_VLC 2.0 h		*cbp1*-1:*CBP1*_VLC 2.0 h vs *cbp1*-1_VLC 2.0 h	
	log_2FC	FDR	log_2FC	FDR	log_2FC	FDR	log_2FC	FDR	log_2FC	FDR	log_2FC	FDR
**Cre01.g000150**	1.34	2.2.E-04	1.78	1.1.E-13	-0.40	4.9.E-01	0.42	3.0.E-01	-0.45	5.4.E-01	0.01	1.0.E+00
**Cre01.g053950**	3.65	2.0.E-34	2.05	1.9.E-19	0.36	4.9.E-01	0.17	7.4.E-01	0.29	7.0.E-01	0.30	8.6.E-01
**Cre03.g151650**	1.37	1.3.E-03	1.78	2.7.E-13	-1.09	3.6.E-03	0.09	8.5.E-01	-1.62	3.3.E-05	-0.21	9.3.E-01
**Cre03.g162800**	2.36	3.6.E-03	3.75	6.7.E-05	-0.12	8.8.E-01	1.94	1.1.E-10	-0.91	2.6.E-01	1.12	1.2.E-01
**Cre03.g212641**	2.30	1.3.E-14	1.44	2.1.E-07	-0.08	8.9.E-01	-0.03	9.6.E-01	1.27	1.0.E-02	0.18	9.5.E-01
**Cre03.g212977**	3.58	3.4.E-26	1.78	2.8.E-12	0.10	8.8.E-01	0.54	2.7.E-01	-0.20	8.1.E-01	0.33	8.9.E-01
**Cre04.g215900**	3.90	1.5.E-22	1.24	1.1.E-05	0.23	7.6.E-01	0.35	4.9.E-01	0.45	4.7.E-01	0.06	9.9.E-01
**Cre04.g215952**	4.69	1.4.E-20	1.44	1.6.E-04	3.67	1.2.E-19	0.68	3.5.E-02	2.71	5.2.E-10	0.28	9.0.E-01
**Cre04.g222750**	2.32	2.2.E-12	1.17	6.0.E-06	0.87	2.3.E-01	1.23	2.9.E-04	-0.09	9.5.E-01	0.33	9.4.E-01
**Cre04.g222800**	1.44	3.2.E-06	1.08	1.4.E-04	0.35	5.5.E-01	1.09	7.6.E-03	-0.91	1.1.E-01	0.63	6.3.E-01
**Cre04.g223100**	2.60	5.6.E-22	5.32	1.5.E-75	-1.23	1.4.E-03	0.88	6.3.E-03	-0.43	5.5.E-01	0.63	6.9.E-01
**Cre06.g278199**	2.92	1.5.E-16	1.08	4.7.E-04	2.97	1.1.E-15	0.60	5.4.E-02	1.92	2.5.E-06	0.93	2.6.E-02
**Cre06.g309000**	2.65	2.6.E-07	3.66	7.4.E-12	0.21	7.6.E-01	1.08	4.9.E-03	-0.39	6.5.E-01	0.64	7.4.E-01
**Cre07.g339000**	2.53	9.2.E-09	1.23	4.0.E-03	0.45	3.4.E-01	0.59	8.6.E-02	0.04	9.6.E-01	0.28	8.6.E-01
**Cre08.g367400**	3.86	5.1.E-15	1.86	1.2.E-06	0.62	2.1.E-01	0.98	4.5.E-03	-0.45	4.7.E-01	0.69	8.0.E-01
**Cre08.g367500**	2.70	3.8.E-07	1.73	4.4.E-05	0.74	1.6.E-01	1.06	6.2.E-04	-0.38	5.8.E-01	0.78	7.2.E-01
**Cre09.g394473**	1.61	4.0.E-06	1.84	5.2.E-12	-0.93	1.9.E-02	-0.15	7.6.E-01	-1.10	8.7.E-03	-0.21	9.6.E-01
**Cre09.g395732**	1.98	3.0.E-09	1.62	8.5.E-09	-0.03	9.6.E-01	-0.14	8.2.E-01	0.81	1.7.E-01	-0.19	9.5.E-01
**Cre09.g399552**	4.20	4.5.E-33	3.81	8.8.E-39	-0.19	7.9.E-01	0.93	4.1.E-02	0.02	9.8.E-01	0.66	2.9.E-01
**Cre10.g452800**	1.39	6.4.E-07	2.14	1.0.E-15	-0.45	3.5.E-01	0.33	4.4.E-01	0.16	8.4.E-01	0.00	1.0.E+00
**Cre11.g481104**	1.50	1.2.E-04	1.02	2.1.E-03	-0.17	7.7.E-01	0.23	6.8.E-01	-0.13	8.6.E-01	0.33	7.7.E-01
**Cre16.g662600**	6.39	1.5.E-38	4.93	7.5.E-42	0.28	7.6.E-01	0.46	3.9.E-01	1.42	2.5.E-02	0.18	9.6.E-01
**Cre16.g663450**	1.15	3.8.E-03	1.70	1.6.E-05	-0.04	9.4.E-01	0.56	9.8.E-02	-0.50	3.8.E-01	0.47	6.6.E-01
**Cre16.g667250**	1.26	8.4.E-05	1.02	4.2.E-03	0.60	2.0.E-01	0.75	2.8.E-02	-0.08	9.2.E-01	0.14	9.9.E-01
**Cre16.g674291**	4.00	3.2.E-42	1.07	1.8.E-05	0.71	9.2.E-02	0.18	6.6.E-01	2.02	8.1.E-08	0.70	6.8.E-02
**Cre16.g685050**	1.54	5.0.E-07	1.99	8.2.E-15	0.23	6.3.E-01	0.60	6.0.E-02	0.58	2.9.E-01	0.54	5.4.E-01
**Cre16.g685100**	1.52	2.5.E-03	2.37	3.0.E-06	0.57	3.5.E-01	1.48	2.3.E-04	0.67	4.4.E-01	0.76	4.3.E-01

Gene IDs represent the gene accession numbers from Phytozome.

Table S7. VLC-inducible and CCM1-dependent genes differently expressed in *cbp1*-1 cells compared with those in WT and *cbp1*-1:*CBP1* cells in VLC conditions

Gene ID	Gene name	Description	Average TPM in WT			Average TPM in *ccm1*-1			Average TPM in *ccm1*-1:*CCM1*			Average TPM in *cbp1*-1			Average TPM in *cbp1*-1:*CBP1*		
			HC	VLC 0.3 h	VLC 2.0 h	HC	VLC 0.3 h	VLC 2.0 h	HC	VLC 0.3 h	VLC 2.0 h	HC	VLC 0.3 h	VLC 2.0 h	HC	VLC 0.3 h	VLC 2.0 h
**Cre01.g047650**	—	F-box protein with leucine-rich repeats protein	1.4	8.6	9.5	1.3	2.0	1.1	1.2	5.5	14.8	4.4	27.0	13.2	3.8	13.7	8.1
**Cre03.g198250**	—	LCI12 paralog	422.8	1134.5	515.9	104.6	136.2	163.9	321.1	800.4	438.6	178.2	226.5	82.5	160.0	384.8	196.3
**Cre03.g204465**	—	GT90 family protein 36	0.2	1.2	5.0	0.1	0.1	0.1	0.2	0.5	4.4	1.3	6.5	9.3	2.0	2.8	6.2
**Cre03.g204577**	***DNJ31***	DnaJ-like protein	0.0	5.8	32.5	0.0	0.0	0.0	0.0	1.5	39.3	0.1	36.7	52.0	0.2	7.4	16.4
**Cre04.g216550**	—	—	0.9	4.7	5.5	1.2	8.9	7.5	0.8	5.2	7.6	2.1	14.3	15.4	1.1	7.3	6.9
**Cre04.g223250**	***LCIE***	Low-CO2 inducible protein E	0.3	7.4	6.4	0.1	0.0	0.0	0.3	2.7	8.2	0.4	49.8	33.1	0.1	9.2	12.0
**Cre05.g245500**	***FAP175***	Ankyrin Repeat Flagellar Associated Protein 175	0.7	6.5	3.7	0.8	1.0	0.5	0.3	3.2	4.0	1.2	2.3	2.4	3.7	10.6	6.5
**Cre08.g380700**	—	—	2.7	5.1	17.0	5.1	4.5	5.6	6.1	6.0	19.9	13.8	22.1	33.8	8.6	11.5	18.7
**Cre11.g469650**	—	—	1.9	4.9	8.2	2.4	1.7	4.2	1.6	3.9	7.9	3.8	14.4	11.6	4.2	6.8	8.4
**Cre12.g527250**	—	—	0.8	5.2	6.6	0.9	1.1	0.7	0.2	1.7	4.6	2.0	23.9	22.7	3.9	9.2	12.6
**Cre12.g555700**	***DNJ15***	DnaJ-like protein	0.1	5.8	83.1	0.0	0.0	0.0	0.0	0.6	96.7	0.2	48.5	110.0	0.1	7.5	43.0
**Cre16.g652200**	***MMP9***	Metalloproteinase of VMP family	3.8	21.3	5.7	2.4	21.4	8.0	6.5	32.1	9.7	1.3	3.2	2.7	2.5	8.6	4.2
**Cre17.g719150**	—	LCI12 paralog	0.3	5.1	7.1	0.1	0.0	0.0	0.4	4.0	4.8	2.2	3.2	0.8	3.6	9.0	10.8
**Cre19.g751047**	—	LCI12 paralog	429.8	1526.0	857.7	71.1	92.8	123.9	351.0	1080.9	717.1	304.9	412.5	155.0	417.1	1093.8	599.2

Gene ID	WT_HC vs *cbp1*-1_HC		*cbp1*-1:*CBP1*_HC vs *cbp1*-1_HC		WT_VLC 0.3 h vs *cbp1*-1_VLC 0.3 h		*cbp1*-1:*CBP1*_VLC 0.3 h vs *cbp1*-1_VLC 0.3 h		WT_VLC 2.0 h vs *cbp1*-1_VLC 2.0 h		*cbp1*-1:*CBP1*_VLC 2.0 h vs *cbp1*-1_VLC 2.0 h	
	log_2FC	FDR	log_2FC	FDR	log_2FC	FDR	log_2FC	FDR	log_2FC	FDR	log_2FC	FDR
**Cre01.g047650**	1.21	2.4.E-03	0.34	7.0.E-01	1.33	1.4.E-03	1.23	3.6.E-04	0.01	9.9.E-01	0.61	5.0.E-01
**Cre03.g198250**	-1.68	7.2.E-09	0.27	7.3.E-01	-2.61	2.9.E-13	-0.50	1.1.E-01	-3.09	9.7.E-16	-1.35	6.0.E-04
**Cre03.g204465**	2.19	1.5.E-04	-0.55	5.1.E-01	2.09	6.6.E-07	1.49	3.9.E-04	0.44	4.0.E-01	0.48	7.9.E-01
**Cre03.g204577**	N.D.	N.D.	N.D.	N.D.	2.34	8.0.E-07	2.56	9.3.E-09	0.21	8.0.E-01	1.57	1.9.E-01
**Cre04.g216550**	0.80	7.2.E-02	1.13	1.2.E-02	1.31	2.1.E-04	1.24	2.4.E-05	1.00	4.6.E-02	1.06	1.6.E-01
**Cre04.g223250**	N.D.	N.D.	N.D.	N.D.	2.44	3.7.E-09	2.69	2.1.E-17	1.88	2.8.E-05	1.37	7.1.E-02
**Cre05.g245500**	0.27	6.3.E-01	-1.50	8.5.E-06	-1.78	1.2.E-06	-1.95	5.0.E-12	-1.10	2.3.E-03	-1.55	8.5.E-08
**Cre08.g380700**	1.91	2.5.E-09	0.79	8.4.E-03	1.90	5.5.E-06	1.25	6.4.E-04	0.52	4.9.E-01	0.76	3.2.E-01
**Cre11.g469650**	0.58	1.3.E-01	-0.03	9.8.E-01	1.25	1.4.E-03	1.31	8.5.E-05	0.06	9.3.E-01	0.37	7.7.E-01
**Cre12.g527250**	0.82	8.7.E-02	-0.85	1.1.E-01	1.85	2.2.E-05	1.60	1.2.E-05	1.31	3.1.E-03	0.76	1.9.E-01
**Cre12.g555700**	N.D.	N.D.	N.D.	N.D.	2.76	4.4.E-10	2.96	1.2.E-17	-0.06	9.4.E-01	1.27	3.6.E-01
**Cre16.g652200**	-1.99	3.6.E-06	-0.86	6.0.E-02	-2.96	2.1.E-15	-1.13	2.1.E-04	-1.46	9.6.E-03	-0.72	3.1.E-01
**Cre17.g719150**	2.26	1.3.E-03	-0.57	5.7.E-01	-0.96	8.6.E-02	-1.22	8.8.E-03	-3.54	2.5.E-07	-3.76	8.4.E-12
**Cre19.g751047**	-0.93	1.6.E-03	-0.34	6.0.E-01	-2.17	1.2.E-09	-1.14	1.0.E-05	-2.92	8.7.E-12	-2.05	2.7.E-06

Gene IDs represent the gene accession numbers from Phytozome.
